# The national survey of academic researchers: New facts and data

**DOI:** 10.1371/journal.pone.0340642

**Published:** 2026-02-19

**Authors:** Kyle Myers, Wei Yang Tham, Jerry Thursby, Marie Thursby, Nina Cohodes, Karim Lakhani, Rachel Mural, Yilun Xu

**Affiliations:** 1 Harvard Business School, Boston, Massachusetts, United States of America; 2 University of Toronto, Toronto, Ontario, Canada; 3 Georgia Institute of Technology, Atlanta, Georgia, United States of America; 4 Harvard Kennedy School, Cambridge, Massachusetts, United States of America; Cardiff University, UNITED KINGDOM OF GREAT BRITAIN AND NORTHERN IRELAND

## Abstract

We introduce a new survey of professors at roughly 150 of the most research-intensive institutions of higher education in the US. We document seven new features of how research-active professors are compensated, how they spend their time, and how they perceive their research pursuits, which we organize under three themes. *Earnings and inequality:* (1) there is more inequality in earnings within fields than there is across fields; (2) institutions, ranks, tasks, and sources of earnings can account for roughly half of the total variation in earnings; (3) there is significant variation across fields in the correlations between earnings and different kinds of research output, but these account for a small amount of earnings variation. *Research productivity and inputs*: (4) measuring professors’ productivity in terms of output-per-year versus output-per-research-hour can yield substantial differences; (5) professors’ beliefs about the riskiness of their research are best predicted by their fundraising intensity, their risk aversion in their personal lives, and the degree to which their research involves generating new hypotheses. *Research output choices:* (6) older and younger professors have very different research outputs and time allocations, but their intended audiences are quite similar; (7) personal risk-taking is highly predictive of professors’ orientation towards applied, commercially relevant research. An anonymized version of the data is publicly available at: https://tny.sh/nsar.

## 1 Introduction

Researchers are at the core of economic growth. The quantity and quality of their new ideas are what enable technological change [1]. In the US, the most common employers of Ph.D.-level researchers are academic institutions; according to the 2021 National Science Foundation Survey of Doctoral Recipients: 42% of Ph.D. scientists and engineers work at educational institutions; 37% work at private, for-profit businesses; 15% work at non-profit or government agencies; and the remainder are employed by other organization types or are self-employed [2]. And a large body of evidence has documented the importance of academic science for industrial progress (e.g., see [3] or [4] for reviews on this topic). Amidst recent concerns of declining R&D productivity [5,6] and an increasing division of labor between industrial firms and professors in academia [7], it has become increasingly important to understand professors’ roles in the scientific workforce.

Once dominated by philosophers (e.g., [8,9]), the “science of science” has become increasingly populated by quantitative analyses by economists, sociologists, physicists, and other scholars looking to turn their empirical toolkits inwards [10,11]. However, most of these empirical studies make use of a small number of data sources that are byproducts of conducting science, which were often not designed as intentional sources of data to study science. Inputs are usually measured using federal grant databases, which cover only a fraction of funding flows and leaves unobserved one of the most important inputs in the scientific production function, researchers’ time. Outputs are usually measured using publication databases, which can generate serious measurement error for any researcher whose output is not codified in print and makes across-field comparisons difficult to interpret given the range in publication norms. Many other important aspects of researchers’ work and lives are left only partly visible at best: their professional position (e.g., tenure status, administrative duties); their incentives (e.g., sources of income); and the objectives of their research (e.g., their intended audience). Still, impressive efforts are still underway to improve the fidelity and interoperability of existing meta-science datasets (e.g., [12,13], UMETRICS at https://iris.isr.umich.edu/, and the Innovation Information Initiative at https://iii.pubpub.org/datasets).

In this paper, we document a new nationally representative survey of research-active professors at roughly 150 of the largest institutions of higher education in the US. The population and sample include professors from all fields of science, broadly construed: engineering, math, and related fields; humanities and related fields; medicine and health; natural sciences; social sciences. The survey instrument includes a number of novel elements related to professors’ rank and tenure status, time use, funding, salaries and sources thereof, the nature of their research, and a battery of socio-demographic and household-related factors. For the majority of our analyses, we focus on professors who report a non-zero amount of their time being spent on research activities (95% of respondents).

An anonymized version of the data is publicly available at: https://tny.sh/nsar.

Our approach follows a long line of prior work that has used surveys of academic researchers to uncover their otherwise unobservable features, choices, preferences, or beliefs (e.g., [14–31,32]). However, unlike many prior surveys of researchers, a guiding principle of our effort was “breadth over depth” such that many design choices reflect an objective of shedding new light on features of this market that have been largely ignored by empiricists. Our hope is that the summary statistics and correlations in this survey will spark more detailed, focused, and rigorous investigations into the causal effects underlying the patterns we see.

Before reporting the major findings of the survey, we describe the population, sampling methodology, recruitment protocol, and summary statistics. To test for representativeness, we use multiple dimensions of data that are observable for respondents and non-respondents. At the institutional level, we use data from the National Science Foundation’s HERD survey [33] to show that respondents come from institutions that receive relatively equal amounts of research funding from a variety of sources. At the individual level, we use data from publication and grant records to show a high degree of similarity in these metrics between respondents and non-respondents. The only key difference between our sample and the population is an under-response from professors at medical schools, which we discuss further below. We sometimes observe other statistically significant degrees of non-response bias, but the practical magnitudes are often relatively small and on the scale of 5 percent of means. Additionally, as one test of attention and honesty, we match a sub-sample of respondents to their publicly reported salaries and find a high degree of alignment.

We organize our results under three broad themes. The first concerns earnings and inequality, documenting how much professors (and their households) earn, the variables that account for those differences, and how professors in different fields are implicitly compensated differently for their observable research output. The second addresses research production inputs, focusing on how aggregate output may sometimes reflect time allocation choices rather than productivity differences, and introducing ex-ante measures of risk. The third highlights research production choices, showing how professors’ intended outputs and audiences vary by career stage and how basic–applied orientation varies. We document seven new findings about the research-active professor workforce:

### 1.1 Earnings and inequality

Finding 1: There is much more inequality in earnings within fields than across. This holds true at the household level because professors exhibit positive assortative matching.

Finding 2: Differences in institutions, faculty ranks, tasks, and sources of earnings can account for roughly half of the variation in earnings across professors.

Finding 3: There are significant differences across fields in the implied payoff to producing observable research output (e.g., earnings per publication). However, research output and payoff differences can only account for a small amount of the variation in earnings across professors.

### 1.2 Research productivity and inputs

Finding 4: Professors with higher gross output (i.e., annual publications) are not always more productive on a per-research-hour basis because of substantial variation in professors’ time allocations. This is especially true for non-tenure-track professors.

Finding 5: Three of the strongest predictors of professors’ beliefs about the riskiness of their research are: (1) the share of their time spent fundraising; (2) their risk-taking in their personal lives; and (3) their orientation towards generating new hypotheses with their research (as opposed to testing hypotheses).

### 1.3 Research output choices

Finding 6: Older professors have different intended research outputs than younger professors (i.e., focusing on books as opposed to journal articles), but their intended audiences are the same. Administrative duties exhibit a rise and fall over professors’ careers, often with a discontinuous increase after receiving tenure, which can explain a large fraction of the decline in research hours post-tenure.

Finding 7: Professors’ position on the basic–applied spectrum can be proxied with the intended output and audience of their research; more applied “Edison-like” professors, whose output is more likely to be tools and products and whose audience is more likely to be businesses and policymakers, report a higher willingness to take risks in their personal lives.

We do not report any causal effects in this paper, taking all equilibrium correlations as being representative of some combination of treatment and selection effects. Furthermore, the data currently exists only as a cross-section. Thus, variation across professors of different ages reflects both temporal dynamics as well as changes to the composition of this workforce.

In some cases, we report decompositions based on the *R*^2^ and partial-*R*^2^ statistics based on simple linear models. Our goal with these exercises is to determine how much the variation in the focal outcome can be described by the covariates, which indicates the extent to which any treatment or selection effects are important along those dimensions. We also report the results of “observational regressions”, a term which we will use to describe regressions of professors’ features on a set of possibly endogenous covariates. Similarly, we sometimes employ ML-based covariate selection methods to identify variables with predictive power (e.g., [34]). The broad patterns that emerge provide useful views of equilibrium relationships and motivate new hypotheses about the incentives facing academic researchers.

We hope the results reported in this paper will spark further investigation into the academic research workforce. The rest of the paper is organized as follows: Sect 2 describes the survey methodology and some summary statistics; Sect 3 walks through our key new findings; Sect 4 concludes with a discussion.

## 2 Methodology: Population, sampling, and survey

### 2.1 Population and sampling

Our target population is US professors who conduct research at major institutions of higher education. We identify this population by selecting the 158 largest institutions in the US per their total R&D funding reported in the National Science Foundation’s 2019 Higher Education R&D (HERD) survey [33]. We hired individuals to manually collect the emails of professors from these universities’ websites. See Appendix A for more. We identified these individuals as people listed on institutions’ websites with the word “professor” in their title, recording their title as well as information on each professor’s name, program and/or department and/or college, and professorial rank. Our requirement of the word “professor” in the title was driven by the logistics of data collection. It is a simple, observable feature to rule identifying individuals in or out of population. Anything beyond this proved too complicated in our data collection process. An important question is to what extent we miss relevant individuals without this moniker. We cannot arrive at a conclusive estimate here since there is no clear definition of relevance with which to benchmark. As one potential benchmark, data from the US National Center of Education Statistics indicates that only a few percentage points of these institutions’ full-time instructional staff are not considered “faculty” [35]. Table 1 reports summary statistics for the institutions included in the population based on variables sourced from websites as well as the HERD survey. Appendix Fig B1 illustrates some joint distributions of fields, institutions, and ranks showing a significant amount of heterogeneity in the organizational structures.

**Table 1:** Institution-level population summary statistics.

	type	count	mean	sd
*prof. share, by rank*				
assistant	[0,1]	159	0.25	0.10
associate	[0,1]	159	0.23	0.06
full	[0,1]	159	0.36	0.11
adjunct, clinical, other	[0,1]	159	0.15	0.11
*prof. share, by field*				
engineering, math & related sciences	[0,1]	159	0.17	0.13
humanities & related sciences	[0,1]	159	0.17	0.10
medicine & health sciences	[0,1]	159	0.36	0.30
social sciences	[0,1]	159	0.13	0.09
natural sciences	[0,1]	159	0.17	0.11
num. prof., total	[0,∞)	159	843.87	554.17
num. schools	[0,∞)	159	11.73	5.74
num. departments	[0,∞)	159	80.27	40.42
*HERD data*				
total R&D funding, M-$	[0,∞]	158	469.55	414.93
federal R&D funding, M-$	[0,∞)	158	249.60	273.42
non-federal R&D funding, M-$	[0,∞)	158	219.96	182.89
total wage bill, M-$	[0,∞)	158	205.98	183.85
num. principal investigators	[0,∞)	158	862.89	729.41

*Note*: All variables are from the NSAR population, except for the last rows, which are sourced from HERD.

Our sampling process was as follows. Based on the information gathered, we classified these emails into one of twenty fields of study and one of four ranks (assistant, associate, full or emeritus, and adjunct or other). We then sent an e-mail invitation to a randomly-selected half of the e-mails within each field-rank cell. These e-mails were distributed from October 2022 to March 2023. Participants gave consent through the survey link before they proceeded with responding to the survey.

The population consisted of 264,036 unique e-mails. We e-mailed a total of 131,672 individuals and 4,388 (3.33%) completed the survey. (This response rate is more than twice what has been obtained from sourcing academic researcher contacts from the corresponding author data contained within the publication record (e.g., [36]).) Our final sample consists of professors from engineering, math and related sciences (737), the natural sciences (680; e.g., biology, chemistry, physics), social sciences (892; e.g., economics, political science, psychology, sociology), humanities and related science (821; e.g., art, history, education, linguistics), and health or medical sciences (1,258; e.g., schools of medicine or public health). These five aggregate groupings of fields were chosen partly based on the results of a principal component analysis to identify fields where professors responded to the survey similarly. Appendix Table B1 reports the results of a single-component PCA based on the entire survey, averaging at the field level. We then aggregated fields together by primarily relying on this score, with some minor adjustments to align with our understanding of these fields. For instance, we group Medical School-based professors with those in other Medicine- or Health-related fields, and we also assign sociology to the aggregate field of social science despite the marked difference in average PCA score for this field. We sometimes use these five aggregate groupings of fields in our discussion and empirical analyses given the small sample sizes within the narrower field definitions.

### 2.2 Potential survey biases

Ideally, our respondents would report all answers accurately and their responses would reflect the preferences and characteristics of the full population. We cannot formally test this, but we can take some steps to investigate the possibility of inattention and non-response bias and, in the case of non-response bias, possibly account for it.

As a test of researchers’ attention and their willingness to report truthfully, we can compare their self-reported salaries to their publicly-reported salaries for the subset of researchers at institutions that make such data public. To do so, we manually traced respondents at 89 institutions with public salary data to their records in these public sources. During this match, we used our data on researchers’ e-mail addresses and institutional affiliations to maximize fidelity of the match. Still, there is likely non-zero measurement error due to both (1) manual errors in the name merging process, and (2) our inability to perfectly confirm that the self- and publicly-reported salaries were referring to the same year of employment. Appendix Fig A3 plots the relationship between these two sources of salary data. The correlation between the two is 0.75, and for roughly 75% of observations the difference between the self- and publicly-reported salary is less than 30%. This suggests the vast majority of respondents are responding truthfully along this dimension.

We use two auxiliary data sources to compare respondents to the population in terms of observable variables. First, we use our internally collected data on professors’ fields and ranks to test for differences between the respondent sample and the population. In Appendix A.3, we show that our respondents are slightly more likely to be full professors and less likely to be adjunct, clinical or other professors compared to the population. We also see a significant under-response from the medical and health sciences relative to other fields. Overall, this suggests that the results reported here may be less generalizable to the full spectrum of professors across medical schools or those in adjunct or clinical professor tracks.

Second, in Appendix A.3, we also compare our respondents to the population according to the HERD survey, which reports on the amounts of R&D funding flowing to each of the institutions in our population. When we examine measures including total funding amounts, funding by source, and by type, we do find some evidence that professors who complete our survey are located at institutions with lower-than-average amounts of funding. However, graphical illustrations and statistical tests of these comparisons show that the difference is relatively small in economic magnitude. We have good representativeness over the full distribution, and the average difference in funding amounts between the institutions of respondents and non-respondents is generally in the range of 4–6%.

We are also able to merge roughly two-thirds of the population to their records in the Dimensions database [37] using a fuzzy, name- and affiliation-based merging process. This allows us to compare individual-level grant input and publication output data across our population and respondents, drawing on all types of publication outputs indexed in Dimensions between 2003 and 2023. This exercise is reported in Appendix A.3. Again, we find economically small differences between the full population and respondents, most of which are not statistically significant. Our respondents’ average publication output rates, field-normalized citation rates, and grant receipt rates and amounts are all within a few percentages of what is observed in the full population. Likewise, graphical investigations show strong overlap in the support of these variables. Overall, our respondent sample appears very similar to the population along many observable dimensions.

In order to ensure that all statistics reported reflect the population, we use inverse probability weights. We construct these weights by regressing an indicator variable for survey completion on a vector of indicator variables for field, professor’s rank, institution, and arms of the participation incentive and reminder experiment they were assigned. We interact field and rank indicators in this regression to capture field-rank specific differences in response rates. We estimate this as a probit regression using data on the full set of professors that we emailed, which was a random 50% subsample of the population. Only 50% of the population was e-mailed based on feedback and requirements from our IRB approval process. The inverse predicted probabilities from this probit regression serve as our weights. All results are qualitatively the same and quantitatively very similar if these weights are not used, which is consistent with our results showing that sample selection appears relatively random.

### 2.3 Sample summary statistics

Table 2 reports summary statistics for the key questions in the survey, which are documented in further detail in Appendix A.2. The majority of respondents are full professors (40%) followed by roughly equal proportions of assistant (25%) and associate professors (25%) with the remainder being adjunct, clinical, or other types of professors (10%). The distribution across aggregate fields is relatively even, although we imposed these field groupings in a way that sought approximate balance in sub-sample sizes. See Appendix Table A2 for the groupings of the twenty narrower fields into these aggregate fields. The classification of fields into these broader groupings is chosen out of simplicity and with an understanding that it would be impossible to please all professors in terms of how the groups are constructed. Most respondents are tenured (57%), with another 21% still on the tenure-track and 22% not on the tenure track at all. The average number of years since tenure for those who are tenured is approximately 15 years. For those not on the tenure track, the average contract length is about 2.5 years, and for those pre-tenure, the average years until their tenure evaluation is about 2.6 years.

**Table 2:** Professor-level sample summary statistics—Professional and socio-demographic.

	type	count	mean	sd
*rank*				
assistant	{0,1}	4,388	0.25	0.43
associate	{0,1}	4,388	0.26	0.44
full	{0,1}	4,388	0.36	0.48
adjunct, clinical, other	{0,1}	4,388	0.12	0.33
*aggregate field*				
engineering, math & related sciences	{0,1}	4,388	0.15	0.36
humanities & related sciences	{0,1}	4,388	0.15	0.36
medicine & health sciences	{0,1}	4,388	0.42	0.49
social sciences	{0,1}	4,388	0.11	0.32
natural sciences	{0,1}	4,388	0.15	0.36
*tenure status*				
not on tenure track	{0,1}	4,388	0.32	0.46
contract length (non tenure track)	[1,∞)	940	2.32	1.90
pre-tenure	{0,1}	4,388	0.19	0.39
years until tenure eval.	[1,∞)	890	2.83	2.16
tenured	{0,1}	4,388	0.50	0.50
years since tenure	(0,∞)	2,426	14.88	11.49
*work hrs., earnings, research funding*				
work hours per week	[1,∞)	4,388	49.40	14.13
work-hrs. share, research	[0,1]	4,388	0.36	0.23
work-hrs. share, fundraising	[0,1]	4,388	0.08	0.11
work-hrs. share, teaching	[0,1]	4,388	0.26	0.20
work-hrs. share, administration	[0,1]	4,388	0.15	0.15
work-hrs. share, clinical	[0,1]	4,388	0.08	0.20
work-hrs. share, other	[0,1]	4,388	0.07	0.13
own annual earnings	[0,∞)	4,388	157,868.56	99,701.20
earnings share, base salary	[0,1]	4,388	0.64	0.37
earnings share, grant-sponsored	[0,1]	4,388	0.17	0.27
earnings share, supplemental	[0,1]	4,388	0.03	0.07
earnings share, other	[0,1]	4,388	0.04	0.13
earnings share, clinical	[0,1]	4,388	0.03	0.13
5-year guaranteed research funding	[0,∞)	4,388	417,155.52	1,014,702.42
5-year fundraising expectations	[0,∞)	4,388	537,951.77	1,005,599.29
*socio-demographics*				
asian	{0,1}	4,315	0.14	0.35
black	{0,1}	4,315	0.03	0.17
hispanic	{0,1}	4,315	0.06	0.23
other	{0,1}	4,315	0.04	0.20
white	{0,1}	4,315	0.77	0.42
US born	{0,1}	4,288	0.72	0.45
non-US born	{0,1}	4,288	0.25	0.44
married or in partnership	{0,1}	4,244	0.84	0.37
num. dependents	{0,1}	4,306	1.02	1.17
household annual earnings	[0,∞)	4,236	265,271.96	162,398.03

*Note*: All variables are from the NSAR sample. Any counts that do not equal the full sample size are due to the question either being not applicable (i.e., the tenure status questions) or were socio-demographic questions where respondents were not forced to answer.

On average, individuals expect to work roughly 50 hours per week, which is nearly eight hours more than the national average for full-time workers [38]. Specifically, respondents are asked to report their expected time use (i.e., total hours per week and allocations across different tasks) over the coming five years. This is done in order to solicit responses that approximate the short-run steady-state of professors’ time use, and was motivated by pilot studies of our time-use questions. Appendix Fig B2 illustrates the distributions of time use across these categories in more detail. The average annual earnings is approximately $150,000 (s.d.=$90,000), which is approximately the 90^*th*^ percentile in the US [39]. Our questions about professors’ earnings also allow for new views into the distribution of earnings sources. For example, see Appendix Fig B4 for the distribution of professors’ total own earnings sourced from grant-sponsorship.

When asked to report how much guaranteed research funding professors expect to have access to over the coming five years (e.g., due to guaranteed funding lines or previous awards), professors report $85,000 per year on average with a relatively large standard deviation ($200,000 per year). Researchers’ expectations about how much they will fundraise beyond those guarantees are of the same order of magnitude and variance as their guarantees. Appendix Fig B3 provides a more detailed view of funding distributions across aggregated fields.

The majority of the individuals report being White (79%), followed by Asian (12%), with these groups being slightly (Whites) and substantially (Asians) over-represented relative to their shares of the full US population ([39]; note: we allowed respondents to report multiple races/ethnicities). The representation of Black, Hispanic, and other ethnicities is relatively low at 3%, 6%, and 4% respectively. Nearly 25% of professors are non-US born. This is nearly double the rate of the full US population [39], which is yet another signal of the importance of immigration for the US research enterprise (e.g., [40]).

The survey also includes a battery of questions related to the nature of professors’ research (see Appendix A.2). First, respondents are asked to rate on a scale of 0 to 10 whether their research is more about generating (0) or testing (10) hypotheses. In addition, respondents are asked to report the intended outputs and audiences for their research. Options for intended output are: publications, books, tools (e.g. data, software, instruments), or practical applications (products, patents, policies). Options for intended audience are: other academics, policymakers, businesses, or the general public. Respondents indicate whether each of these options is their intended audience or output “Never or rarely”, “Sometimes”, or “Most or all of the time”. Table 3 reports the summary statistics for these variables describing the nature of professors’ research.

**Table 3:** Professor-level sample summary statistics—Nature of research.

	type	count	mean	sd
*intended output*				
journal articles	{0,1,2}	4,388	1.75	0.58
books	{0,1,2}	4,388	0.46	0.66
research materials, tools, etc.	{0,1,2}	4,388	0.67	0.68
products	{0,1,2}	4,388	0.46	0.63
*intended audience*				
academics	{0,1,2}	4,388	1.75	0.58
policymakers	{0,1,2}	4,388	0.76	0.69
businesses and organizations	{0,1,2}	4,388	0.52	0.62
general public	{0,1,2}	4,388	0.77	0.64
*other*				
objective is to test				
(vs. generate) hypotheses	[0,10]	4,388	4.60	2.71
risk of research	[0,10]	4,388	4.33	2.45

*Note*: All variables are from the NSAR sample. The variables of type “{0,1,2}” are conversions of a three-point likert scale increasing in intensity. The variables of type “[0,10]” were solicited directly as real numbers on a scale from 0 to 10.

## 3 Findings

### 3.1 Earnings and inequality

Finding 1: *There is much more inequality in earnings within fields than across. This holds true at the household level because professors exhibit positive assortative matching.*

Variation within and across fields in academic researchers’ earnings is a regular conversation topic within the halls of most universities. But the difficulties of systematically matching researchers’ fields to their earnings have limited investigations into this variation except in specific cases (e.g., [41–45]). For some prior work on professors’ earnings, with results that are relevant to this and other findings in this paper, see: [41,46–53]. Furthermore, most existing data on researchers’ earnings leave the remainder of the household (e.g., spouses’ earnings) untouched despite the importance of the household as an economic unit [54]. We’re referring to pre-tax annual earnings from any source here, decomposing the sources of earnings below. For simplicity, the survey question that solicited earnings did not belabor the distinction between “earned” and “non-earned” incomes, which leaves open the possibility that some respondents reported non-earned income. Still, such income likely would’ve been reported via the “other” source category, which accounts for only 5% of reported earnings on average.

**Fig 1:**
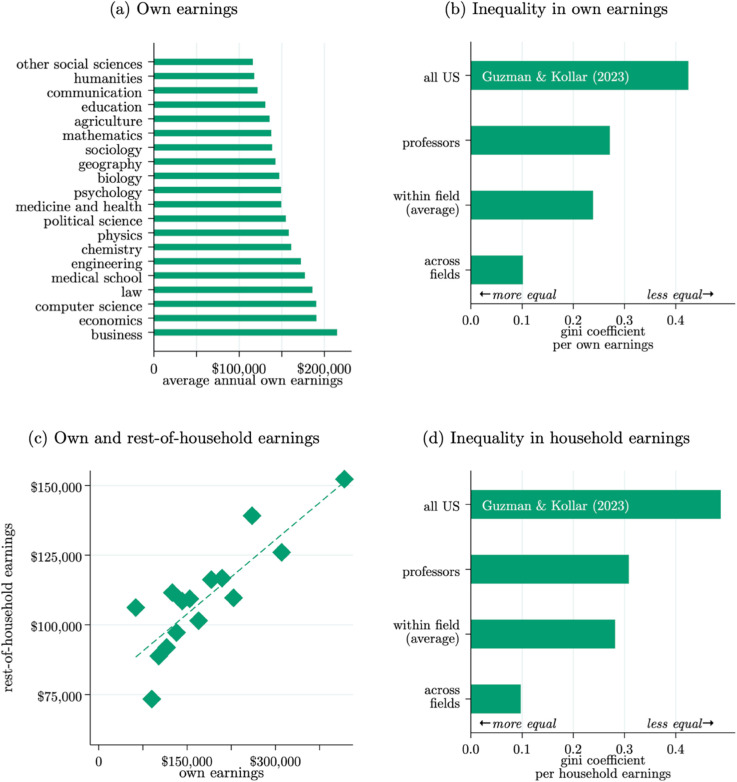
Individual and household earnings across fields. *Note*: Based on 4,236 observations reporting both own and household earnings; Panel (c) is based only on 3,474 professors who report having a partner in their household; the correlation is $0.13^{***}$. All averages reported are post-shrinkage to adjust for differences in sub-sample sizes across fields. Panels (b) and (d) includes estimates from the US Census Bureau [56].

Fig 1 Panel (a) reports field-level average self-reported total annual earnings. Fig 1 reports post-shrinkage means using the empirical Bayes shrinkage methodology of [55] in order to adjust for differences in sub-sample sizes across fields. Professors’ individual earnings span lower values of approximately $130,000 per year in fields such as the humanities, communication, agriculture, and education. Earnings in the highest-paying fields, economics, medicine, law, and business are roughly $200,000 per year.

How much variation in earnings do these field-level averages hide? To get a sense as to the variation in earnings both across and within fields, Panel (b) of Fig 1 plots three alternative individual-level Gini coefficients for the sample and, for comparison, the Gini coefficient for the full US population of full-time workers per recent estimates from the US Census Bureau [56]. The overall Gini coefficient for professors’ own earnings is roughly 0.27, which is significantly lower than the 0.43 observed across all US workers [56] and is comparable to countries such as Belgium, the Netherlands, and Iceland [57]. Appendix Fig B5 reports additional views of earnings variation. Interestingly, there is very little variation in the within-field Gini coefficient across fields, and there is no significant relationship between field-level average earnings and within-field inequality.

To compare inequality in earnings within fields, we estimate a separate Gini coefficient for each field and report the average of these within-field estimates. To compare inequality in earnings across fields, we take the average salary within each field (held constant at associate professor rank) and calculate the Gini coefficient assuming there is one representative professor in each field who earns this (average) amount. Inequality in professors’ earnings is much higher when focusing on the within-field variation (average Gini coef.=0.24, s.d.=0.02) compared to the variation in field-level averages (Gini coef.=0.10).

As evidenced by the strong positive relationship between individual and rest-of-household earnings for those with partners (Fig 1 Panel c), there is clear evidence of positive assortative matching among research professors. Amongst professors with partners, we estimate that each additional $10 of a professor’s own earnings is associated with approximately $1.5 additional dollars in rest-of-household earnings. To illustrate the role of this assortative matching (and the role of multi-earner households more generally) on household-level earnings variation, Fig 1 Panel (d) reports the same Gini coefficients as Panel (b), this time based on household-level earnings. As expected given the positive matching, the pattern remains the same. Compared to the US population of households (Gini coef.=0.49), earnings across professors’ households is more equal (Gini coef.=0.31) with most of the variation driven by earnings differences within fields. The differences between household- and individual-level Gini coefficients in the full US population and professors is relatively similar on the scale of 5 p.p. and 15%. This suggests the positive assortative matching observed amongst professors is similar to that observed in the full population [58].

There is much more inequality in earnings within fields than there is across fields, a fact that is of both inherent and policy relevance to the market for academic research labor. In many of the following analyses, we attempt to identify some of the sources of this variation based on how professors spend their time and what outputs they produce.

Finding 2: *Differences in institutions, faculty ranks, tasks, and sources of earnings can account for roughly half of the variation in earnings across professors.*

Understanding the different incentives researchers face is key to understanding how they allocate their time. How much of the variation in professors’ earnings can be explained by observable differences in their work? The rarity of jointly observing professors’ time use across their many tasks alongside their earnings has limited our ability to investigate these issues. There are numerous investigations into the determinants of professors’ salaries (e.g., [59–63]). However, most of these analyses are limited to a single scientific field and/or cannot specifically isolate inputs (e.g., time allocations) and outputs (e.g., articles published) of professors’ work. In Finding #2 here, we focus on these inputs, and in the next finding we focus on their (research-oriented) outputs. As evidenced in Appendix Figs B1 and B6, there is considerable heterogeneity in professors’ ranks, in how they spend their time, and in the sources of their earnings.

**Fig 2:**
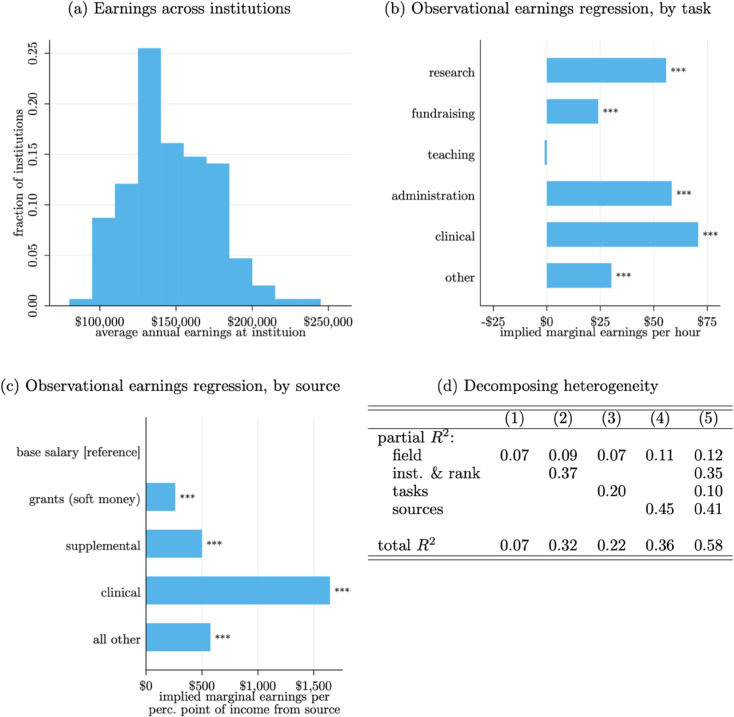
Earnings per institutions, ranks, tasks, and sources. *Note*: Based on 4,388 observations. Observational earnings regressions are of the form: $Y_{i}=\alpha +\sum_{j}X_{i}^{j}\beta^{j}+\epsilon_{i}$, where *Y*_*i*_ is professor *i*’s earnings and $X_{i}^{j}$ is the vector of either (Panel b) the professor’s hours spent on each tasks indexed by *j*, or (Panel c) the percentage points of the professor’s income due to each source indexed by *j*; the bar graphs in Panels (b–c) report the resulting estimates of the $\beta^{j}$ coefficients; stars indicate statistical significance:   *p*<0.1, ^**^ *p*<0.05, ^***^ *p*<0.01. In Panel (c), since the source percentages sum to 1, the base salary category is set to be the reference group. Institutional averages reported are post-shrinkage to adjust for differences in sub-sample sizes across institutions.

To better understand earnings differences, we first estimate rank- and institution-specific average earnings. The average annual earnings by rank are as follows: assistant, 138,409 (s.d.= 78,690); associate, 146,075 (s.d.= 78,125); full, 205,491 (s.d.= 106,219); other, 85,885 (s.d.= 94,284). Fig 2 Panel (a) shows that there is considerable variation in average earnings across institutions. There is some right skew to this distribution, but it is much less skewed than the distribution of firm-level average earnings in the US economy. Here, the 75^*th*^:25^*th*^ percentile ratio is roughly 1.3 and the 90^*th*^:10^*th*^ percentile ratio is roughly 1.8. In the broader economy, these ratios tend to be closer to 2 and 5, respectively [64]. Notably, these are not adjusted for any observable differences across professors.

Next, we estimate observational regressions that relate professors’ earnings to different features of their work. First, we regress their earnings on their time spent on six different tasks (research, fundraising, teaching, administration, clinical, or other). Next, we regress their total earnings on the source of their earnings (base salaries, grant-covered, supplemental earnings from their primary institution, clinical work, or other). These regressions are of the form: $Y_{i}=\alpha +\sum_{j}X_{i}^{j}\beta^{j}+\epsilon_{i}$, where *Y*_*i*_ is professor *i*’s earnings and $X_{i}^{j}$ is the vector of either the professor’s hours spent on each task, indexed by *j*, or the percentage points of the professor’s earnings due to each source indexed by *j*. The results of these two regressions (the estimates of $\beta^{j}$) represent the implied marginal wages of each task (or income source) holding the amount of time spent on the other tasks (or share of income from other sources) fixed. Fig B6 illustrates the variation in these metrics across fields.

There are substantial differences in the implied returns to different tasks (Fig 2, Panel b). Clinical work is associated with the most earnings, nearly $75 per hour. This is to be expected since most “clinical” work performed by professors involves medical care delivery at academic medical systems. Research, fundraising, administration, and “other” tasks have implied marginal wages of roughly $25–50 per hour. Additional time spent on teaching activities has no statistically significant association with earnings (and the point estimate is negative). Clearly, this partly reflects a selection effect whereby positions with larger teaching responsibilities also have lower salaries. Still, this observational regression makes the importance of this selection effect very clear.

Overall, Fig 2 Panel (c) echoes the findings of Panel (b). In short, professors that have a larger fraction of their earnings coming from sources besides their base salaries have larger earnings levels. This is especially true for those undertaking clinical work.

We decompose the variation in earnings across professors more formally in Fig 2 Panel (d). Institution and rank averages explain a considerable amount of within-field variation, as do professors’ tasks and earnings sources. Field-level averages alone describe roughly 8% of earnings variation (Column 1), with the full set of covariates explaining roughly 58% of earnings variation (Column 5). This suggests that upwards of half of earnings variation could be due to these institutional and position-specific features of professors’ work and how compensation varies across these dimensions. It also implies that nearly half of the total variation may be due to other factors or idiosyncratic differences. We focus on one of those possible factors, research output, next.

Finding 3: *There are significant di!erences across fields in the implied payoff to producing observable research output (e.g., earnings per publication). However, research output and payoff differences can only account for a small amount of the variation in earnings across professors.*

How do different fields implicitly reward different types of research progress? And how much of the earnings variation across fields is due to differences in research output or the different way that output is rewarded? For a narrow look at these questions within the field of marketing, see [65]. With approximately three quarters of our sample matched to their grant and publication histories, we can explore these questions further. See Appendix A.6 for more on this matching process and comparisons of the matched and un-matched sub-samples. All metrics are based on output from 2003–2023.

**Fig 3:**
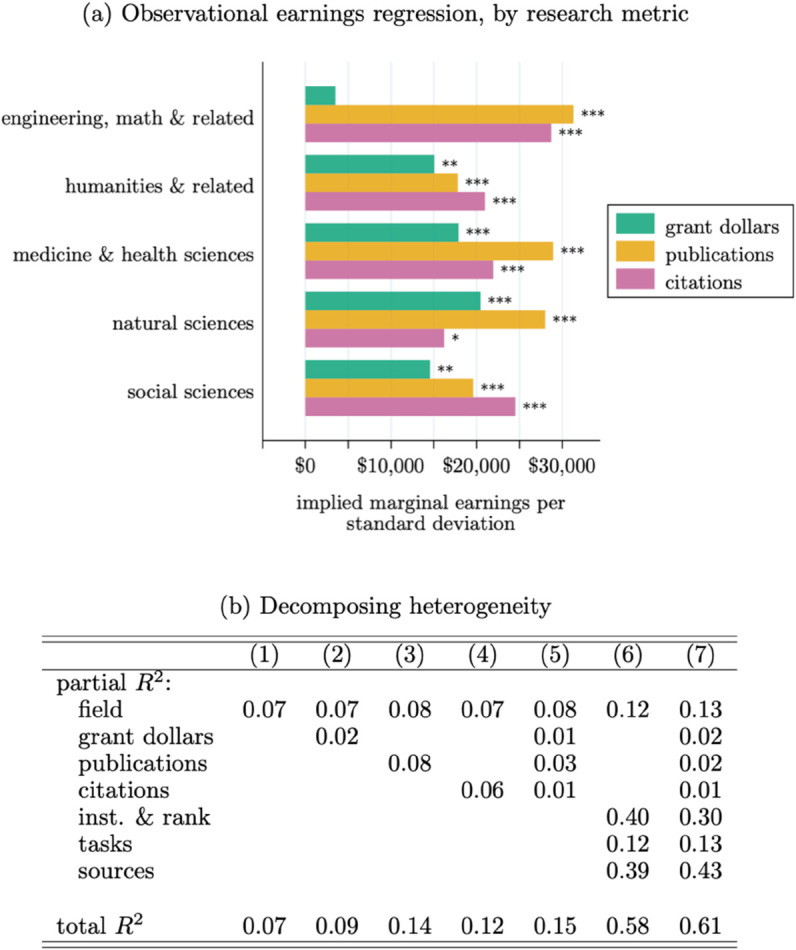
Earnings per research metrics. *Note*: Based on 3,323 observations matched to the publication/grant database. Observational earnings regression is of the form: $Y_{i}=\alpha_{f(i)}+X_{i}\beta_{f(i)}+\epsilon_{i}$, where *Y*_*i*_ is professor *i*’s earnings and *X*_*i*_ is a variable or vector of their standardized inputs and outputs where *f* indexes fields; the bar graph in Panel (a) reports the estimates from univariate regressions including only one metric at a time; the regressions in Panel (b) are based on including all metrics (and other covariates); stars indicate statistical significance:   *p*<0.1, ^**^ *p*<0.05, ^***^ *p*<0.01.

Fig 3 Panel (a) reports the results of a univariate observational regressions of earnings on research inputs and outputs. To allow for heterogeneous returns across fields, we interact the research metrics with indicators for each of the five aggregate fields in our sample. For each of the three metrics, we standardize the variation within each field so that a unit increase in each metric corresponds to a one s.d. increase per the distribution within the field. Within each aggregate field and on an annual basis, one standard deviation in each metric is equivalent to: Engineering and related—$695,072 in funding, 4 publications, and 36 citations; Humanities and related—$146,807 in funding, 2 publications, and 20 citations; Medicine and health—$503,421 in funding, 4 publications, and 47 citations; Natural sciences—$363,639 in funding, 5 publications, and 74 citations; Social sciences—$441,393 in funding, 2 publications, and 24 citations.

We find significant differences across fields in terms of the implied earnings per research metric. Medical and natural sciences are the two fields that appear to implicitly reward grant funding, with a one s.d. increase being associated with $5,000 and $20,000 in additional annual earnings, respectively. We estimate similarly-sized correlations for the humanities and social sciences, but there the relationships are not statistically significant.

In terms of the implied returns to research output, there appears to be two norms: rewards for publications or citations. In the humanities and social sciences, earnings are most clearly correlated with citation-based measures of output. In the other fields, earnings are much more closely connected to publication counts, with citation counts (conditional on publication counts) showing no clear relationship with earnings.

We again decompose earnings heterogeneity, this time focusing on these research metrics, in Fig 3 Panel (b). Recall, this is based only on observations matched to the Dimensions data, which can lead to some discrepancies if compared to Fig 2 Panel (d). Without accounting for any other covariates, differences in these research metrics appear roughly equally as important as field-level differences, with both explaining approximately 10% of earnings variation. However, when we include the full set of institution, faculty rank, task, and earnings source covariates that we explored in Fig 2 (Columns 6–7), we find research output to be much less important. Conditional on these other covariates, research metrics can account for only 3%(=60%-57%) of earnings variation.

When paired with the prior finding, it appears that the traditional metrics of research output often used in the science of science are much less related to professors’ earnings than other attributes of their job. This doesn’t necessarily imply anything about the validity of these metrics as indicators of scientific progress. Nor does this imply anything about the optimality of researchers’ payoffs from conducting research. However, it illustrates that there is potentially a large gap between the way in which professors are financially compensated and the way in which scholars in the science of science field might characterize their performance. We turn more specifically to the notion of research productivity next.

### 3.2 Research productivity and inputs

Finding 4: *Professors with higher gross output (i.e., annual publications) are not always more productive on a per-research-hour basis because of substantial variation in professors’ time allocations. This is especially true for non-tenuretrack professors.*

Despite time being a key scientific input, the difficulty of observing even a proxy of researchers’ time allocations has severely limited our understanding of the labor component of the scientific production function. There have long been studies of higher education focused on professors’ time allocations, with particular focus on the “research-teaching nexus” as it is often referred (e.g., [66–72]). But this work has focused less on analyzing professors scientific productivity per se. This has led many meta-science analyses to assume that researchers all have access to the same amount of time per year and either explicitly or implicitly use researchers’ gross output per year as a measure of productivity.

But of course, professors generally balance multiple roles in a university, only one of them being a researcher. Teaching and advising responsibilities, administrative duties, and grant-writing tasks can all loom large. For example, in grant-intensive fields, many have raised concerns that scientists devote too much time to unproductive activities in order to win grants [73,74]; however, it is difficult to estimate the social value of these efforts since not all time and effort devoted to fundraising is necessarily wasteful [75,76]. Table B2 reports the pairwise correlations for the main categories of time allocation we focus on: research (including supervising others); fundraising for research; teaching or advising (not as a part of their own research); clinical or medical practice; all other activities. We ask respondents to forecast their weekly hours they will spend on each of these activities over the coming 5-year horizon in hopes of them estimating something close to their steady-state time allocations that are not driven by year-to-year idiosyncrasies. Except in the case of fundraising time, which appears to be a partial complement to research time, time spent on all other tasks is associated with a decline in time spent on research.

The key question is whether these other time constraints are allocated in a way that is correlated with researchers’ actual scientific productivity. If time constraints (e.g., administrative duties) are often allocated to researchers with lower hourly scientific productivity (i.e., the two are negatively correlated), then researchers’ annual and hourly output will be very closely aligned. However, to the extent certain researchers with high hourly productivity have fewer opportunities to conduct research because they face additional time constraints, then the alignment between annual and hourly productivity will begin to deteriorate. If the positive correlation between hourly productivity and time constraints was large enough, there could feasibly be no correlation between annual and hourly research output.

**Fig 4:**
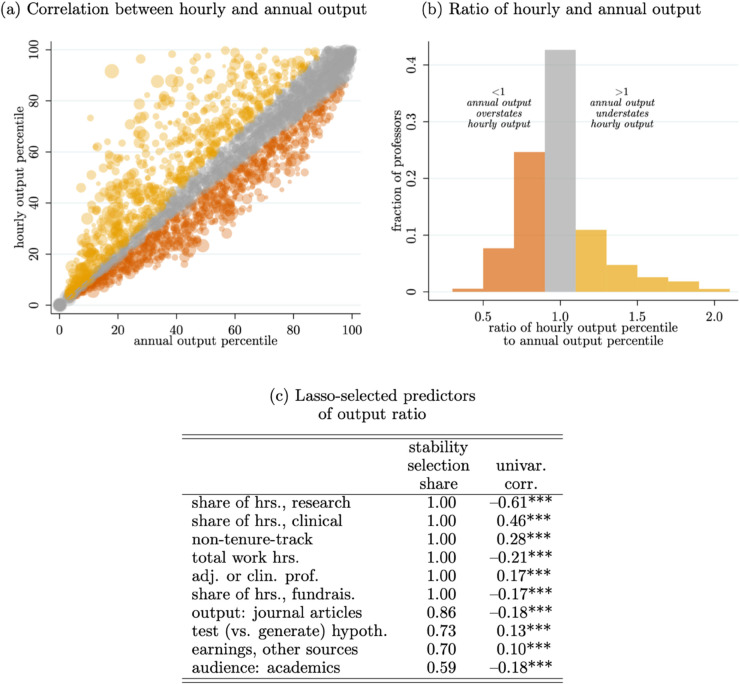
Research productivity and time allocations. *Note*: Based on 3,053 observations reporting non-zero research hours and matched to the publication database. Output is based on field-normalized citation-weighted publication rates per year or per research-hour. In Panel (c), stars indicate the significance of the unconditional correlation:   *p*<0.1, ^**^ *p*<0.05, ^***^ *p*<0.01.

In order to understand how well traditional measures of gross output per year correlate with measures that account for differences in input levels (i.e., output per hour worked), we again focus on the Dimensions-matched sample where we can see professors’ publication output. We calculate their publication output on both an annual and hourly basis (per their field-normalized publication counts). We caveat that our approach here measures productivity per hour by dividing past output using future expected time allocation, thus leading to some measurement error. However, we also expect some persistence in how researchers allocate their time, and the systematic patterns we observe (e.g., non-tenure-track faculty appearing more productive per hour than their total output would suggest) suggest that genuine differences in time constraints are driving results. The correlation between the two measures is *ρ*=0.69, which suggests that annual output is indeed informative of hourly productivity, but it may be misleading for some. To get a better view, Fig 4 Panel (a) plots each individual’s percentile of annual output on the *x*–axis and their percentile of hourly output on the *y*-axis, noting that this compression into percentiles hides the skewed nature of these measures. Researchers below the 45^*o*^ line have an annual output that overstates their hourly output, and vice versa.

To further investigate this issue, Fig 4 Panel (b) plots a histogram of the ratio of each professor’s percentile on an hourly basis compared to the same on an annual basis. We find about 40% of professors have an annual output percentile within 10% of their hourly output percentile (see: the grey shaded bar in the histogram). Another 35% have output percentiles within 20% of each other. There are a significant number of researchers for whom annual output is not a strong proxy for their hourly output. Understanding who these researchers are and whether more resources ought to be allocated to them is a key policy question. Fig B7 shows that the same general pattern holds when focusing on professors’ fundraising productivity (i.e., grant dollars per year or per fundraising-hour).

As a first look, we use the stability selection method of [34] to identify “important” predictors of the gap between researchers’ annual and hourly output (per percentiles). This method proceeds as follows: (1) a random 50% sub-sample is drawn; (2) a standard cross-validation Lasso is used to select the relevant covariates (we use the standard *k*-fold cross-validation lasso program in Stata with all default options); (3) Steps (1–2) are repeated 100 times, recording the share of samples each covariate is selected by the Lasso (i.e., the stability selection share). Fig 4 Panel (c) reports the stability selection share for the top ten covariates along with the correlation between each covariate and the ratio of researchers’ annual and hourly output. Notably, we include field fixed effects as potential controls here. Unsurprisingly, the strongest predictors are variables related to researchers’ work hours. Individuals with the most understated hourly productivity are in non-tenure-track, adjunct, or other positions presumably because they have the largest constraints on their time. Interestingly, there is some evidence that researchers pursuing non-traditional research outputs (that is, not journal articles intended for academics) also appear to have understated hourly productivities. This again may be due to their time allocations focusing more on non-research-specific tasks. Overall, this new view of researchers’ time indicates that gross output measures like publications per year may not provide an unbiased view into researchers’ true underlying productivity in terms of their ability to convert their actual *research* time into scientific output.

Finding 5: *Three of the strongest predictors of professors’ beliefs about the riskiness of their research are: (1) the share of their time spent fundraising; (2) their risk-taking in their personal lives; and (3) their orientation towards generating new hypotheses with their research (as opposed to testing hypotheses).*

Discourse about innovation and science policy often asserts that the system overly discourages scientists from taking risks, causing society to miss out on high-impact scientific discoveries and inventions (e.g. [77]). Most empirical work has focused on this issue relies on ex-post measures of risk-taking based on bibliometric measures. For example, [78–80]. See [81] for an effort that includes a wide range of proxies for concepts underlying risk and novelty, and see [82,83], or [84] alternative approaches. These ex-post measures of publications are clearly limited in their ability to proxy for ex-ante risk-taking by researchers.

To provide a new, alternative view of risk in science, the survey solicits professors’ subjective beliefs about their own risk-taking behaviors. Constructing any sort of field-agnostic (relatively) objective measure of risk-taking that mirrored those commonly used in lab experiments (e.g., gambles over outcomes) proved extremely difficult in pilot tests due to the heterogeneity in relevant outcomes. Hence, our more subjective, but much simpler measure. Using questions structured in the same format as the more general risk preference questions of [85], researchers report how risky they think their own research is, as well as how risky they think their peers think their research is (on a scale from 0 to 10, with larger values indicating more risk). Pilot interviews with scientists suggested that both approaches would prove useful avenues for soliciting researchers’ beliefs. Our preferred metric reported throughout this paper is the average of these two responses, which hopefully serves to reduce some of the measurement error inherent to either phrasing of the question. Fig B8 reports the distribution of this (averaged) risk score, which illustrates significant support across the full range of possible values except for the uppermost tail of risk-taking.

**Fig 5:**
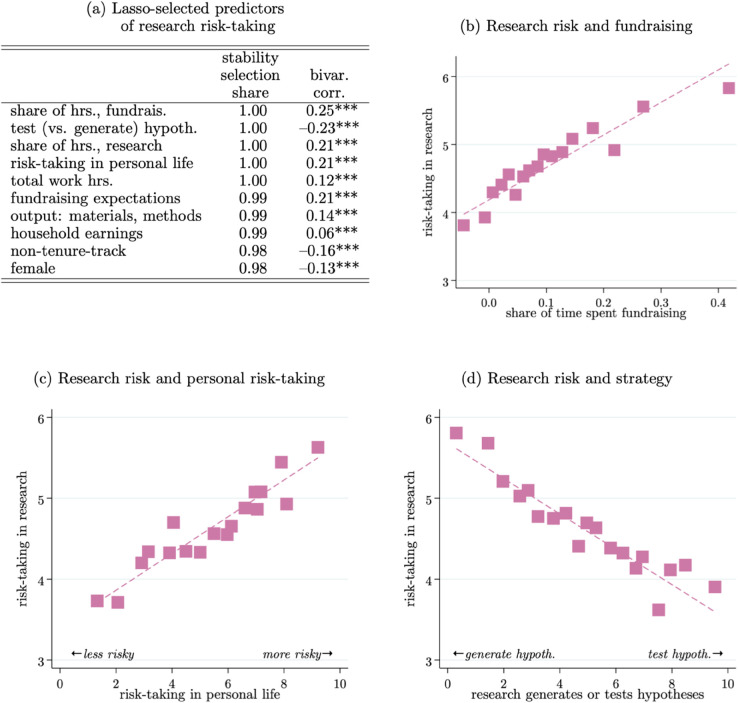
Correlates of research risk-taking. *Note*: Based on 4,186 observations reporting non-zero research hours. Panel (a) reports the results from lasso regressions predicting research risk with the full set of covariates from the sample; the stability selection share column reports the share of 100 bootstrap sub-samples that the covariate is selected in, and the bivariate correlation column reports that correlation for the full sample;   *p*<0.1, ^**^ *p*<0.05, ^***^ *p*<0.01. Panels (b–d) show binned scatterplots relating researchers’ perceptions of the riskiness of their research (*y*-axis) to select covariates (*x*-axis) including field fixed effects to account for field-specific factors; the correlations illustrated in Panels (b–d) are $0.25^{***}$ , $0.21^{***}$, and $-0.23^{***}$, respectively.

We use the ML-based approach of stability selection to identify the covariates that best predict researchers’ perceptions about the riskiness of their research. Fig 5 Panel (a) reports the results of this exercise, with Panels (b–d) showing binned scatterplots of researchers’ risk perceptions based on three of the top predictors we identify via stability selection: (Panel b) the share of time researchers spend on fundraising; (Panel c) researchers’ willingness to take risks in their personal lives; and (Panel d) researchers’ orientation towards generating (as opposed to testing) hypotheses. When constructing these scatterplots, we absorb field fixed effects to remove across-field variation that may be due to idiosyncratic features of fields. Without these fixed effects we obtain very similar results, which indicates that the covariance between risk and these variables is a common occurrence within all fields.

Fig 5 Panel (b) suggests that professors who undertake more fundraising may inherently perceive more risk in their research. This is interesting because “fundraising risk” has not traditionally received much attention due to the difficulties of observing professors’ specific funding streams. This may be driven by the fact that professors who spend a large fraction of their time fundraising tend to be in “soft-money” positions where a portion of their salary is derived from their fundraising. Every percentage point increase in the share of researchers’ time spent on fundraising is associated with an additional $1,095 (s.e.=$58) in annual earnings. Alternatively, it may be that projects that are more ambitious and risky tend to be projects that also require more resources and hence more fundraising. This points to the need for more work on understanding what risk means to researchers (e.g., [86]): What are the outcomes they care about? How do they perceive risk?

Risk-taking in personal life is another strong predictor of researchers’ perceptions of the riskiness in their science. Of course, the simplest explanation here is a survey response bias whereby individuals inflate their risk-taking in both questions and this generates the correlation. However, the pattern is also consistent with researchers’ latent risk-aversion being a key determinant of how they pursue their science. To the extent this is true, it suggests that understanding the extensive margin of selection into science and how it may screen individuals with higher or lower levels of latent risk-aversion would be a fruitful avenue for future research.

Lastly, we see that researchers who report focusing on generating new hypotheses also tend to report higher perceived risk in their research. This aligns with the idea that it is inherently more difficult to capture the value of a good question compared to a good answer; in other words, hypotheses themselves have stronger public-good attributes than tests of hypotheses. Undertaking projects where your ability to capture the value of your efforts is less certain would likely be perceived as more risky. We dig further into researchers’ strategies next.

### 3.3 Research output choices

Finding 6: *Older professors have di!erent intended research outputs than younger professors (i.e., focusing on books as opposed to journal articles), but their intended audiences are the same. Administrative duties exhibit a rise and fall over professors’ careers, often with a discontinuous increase after receiving tenure, which can explain a large fraction of the decline in research hours post-tenure.*

Besides taking (or avoiding) risks, what exactly are research professors intending to do with their research? Here, we dig into our questions related to the intended outputs and audience of professors’ research (see Table 3). All of the questions related to professors’ intended output and audience are solicited on a Likert scale with three values of frequency (“Rarely”, “Sometimes”, “Most of the time”), which we convert into a variable valued {0,1,2} (see Table 3). These measures are intended to reflect the underlying *share* of professors’ scientific production that is destined for a particular output or audience type. Thus, for all of the following analyses, we assume that professors have a fixed level of intentions in these two dimensions and re-scale each of their responses into fractions that the sum to one for each dimension. For example, if a professor reports that all four output types are their intentions “Most of the time”, then we assume that the share of their intended output of each type is 1/4. Specifically, motivated by early work in the economics of science related to life-cycle effects [14], we focus on temporal changes across ages and professional experience. An important caveat to reiterate here is that the cross-sectional nature of the survey means that any temporal dynamics reflect both age- or experience-related effects in addition to any selection effects that occur over the life cycle and/or career cycle.

**Fig 6:**
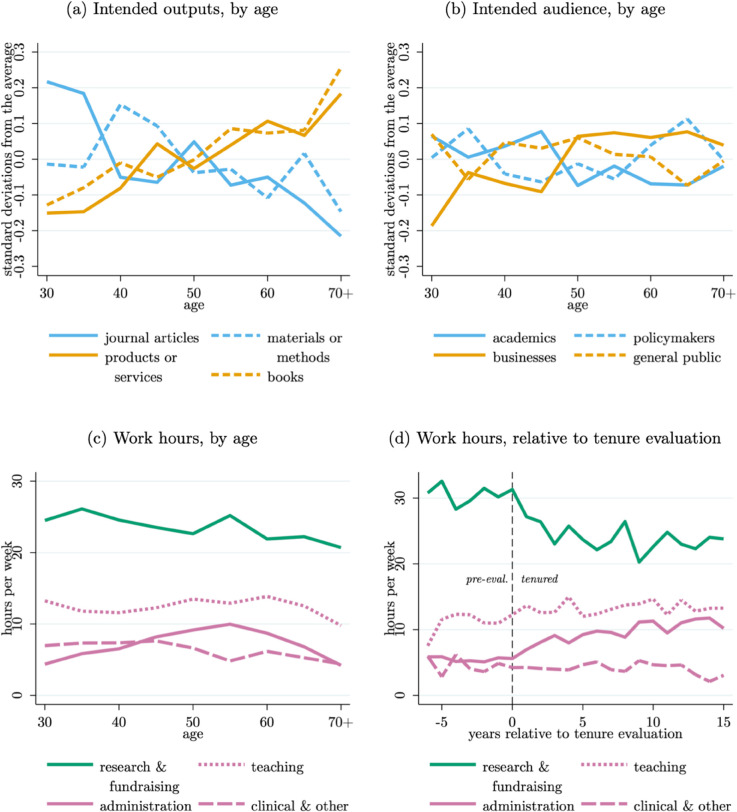
Nature of research, lifecycle, and tenure. *Note*: Panels (a–c) are based on 4,095 observations reporting non-zero research hours and their age; Panel (d) is based on 2,180 observations from tenure-track professors at most 6 years before, or 15 years after, their tenure evaluation and includes only tenured professors post-evaluation. See the main text for details on how the intended output and audience scales are constructed.

Fig 6 Panels (a–b) plot professors’ intended outputs and audiences across the forty years of ages in our sample. Notably, there is a marked evolution in *what* professors are focusing on producing, shifting from a focus on journal articles, materials, or methods in their early decades into a focus on books, products, or services in their later decades (Panel a). In Appendix Table B3, we report regression results of these age-output relationships, which include field fixed effects and are statistically significant.

However, there is no significant change in *who* professors are focusing their efforts towards (Panel b). In Appendix Table B3, we report regression results of these age-audience relationships, which are generally not statistically significant. This pattern suggests that professors’ preferences over their audience are quite stable, but the optimal way of reaching this audience is not. Books as a scientific output have not received much attention by the science of science community, likely because of data limitations. Exceptions include [87–89], and in a more general sense [90].

Fig 6 Panels (c–d) revisit professors’ time use, now looking over the life cycle (Panel c) and more narrowly around the tenure evaluation process for those on the tenure track (Panel d). Here, we group research and fundraising time given their positive correlation as shown in the pairwise time-use correlations of Table B2. As most experienced professors can attest, age and experience are associated with a clear increase in administrative duties. However, this provides one of the first views of this shift in task composition that allows us to quantify the relative increase in administrative duties relative to the change in professors’ time spent on their research. A common result publicized by studies that can observe only publication output across researchers’ careers is the marked decline in their publication output after their early years of work, and especially after receiving tenure [80].

Focusing specifically on Panel (d), we can see that the receipt of tenure is associated with a marked increase in administrative duties and, to a much lesser degree, some increases in teaching and other duties. Aggregating these changes together indicates that, in the first ten years post tenure, approximately 80% of the decline in research and fundraising efforts can be explained by the increase in teaching, administrative, and other effort. In Appendix Table B4, we report regression results from estimating the mean difference in hours worked between pre- and post-tenure professors. We find that tenured researchers spent roughly 5.5 fewer hours per week on research and fundraising, and they spend roughly 3.7 more hours per week on administration. Here again, much like our prior finding on the differences between annual and hourly output, our ability to observe researchers’ time allocations indicates that the “post-tenure glut” in publication may not be any change in productivity on a publication-per-research-hour basis but, to a large degree, may more simply be a decline in input levels. This distinction is important because it speaks to the trade-offs of the institutions of academic science and professors’ responsibilities therein. For instance, the ideal distribution of professors’ administrative duties over the course of their career will depend on, among other things, how their productivity evolves over their life-cycle, which may follow field-specific patterns [14,91,92].

Finding 7: *Professors’ position on the basic–applied spectrum can be proxied with the intended output and audience of their research; more applied “Edison-like” professors, whose output is more likely to be tools and products and whose audience is more likely to be businesses and policymakers, report a higher willingness to take risks in their personal lives.*

Understanding the selection of research topics by professors and the rewards for these choices is crucial to understanding the direction of science. See [93] for an early investigation into the differences in research outputs across fields of science. More specifically related to the finding here, see [19] and [23] for work on scientists’ “taste” for commercially-oriented science. One of the most common approaches to characterizing research is on a spectrum of “basic” to “applied” (e.g., [29,94,95]). However, creating a quantitative measure solely based on existing data is challenging. For example, bibliometric measures such as patent citations may have limited validity in some fields where patenting is rare. To provide an alternative view, the survey includes multiple questions related to the nature of professors’ research (see Table 3). Each question was designed to both capture different types of scientific outputs and audiences, but also to reflect different dimensions of the basic–applied spectrum as often described. See [96] for another survey-based approach that more directly attempts to solicit researchers’ position on this spectrum, which yields findings complementary to ours here.

**Fig 7:**
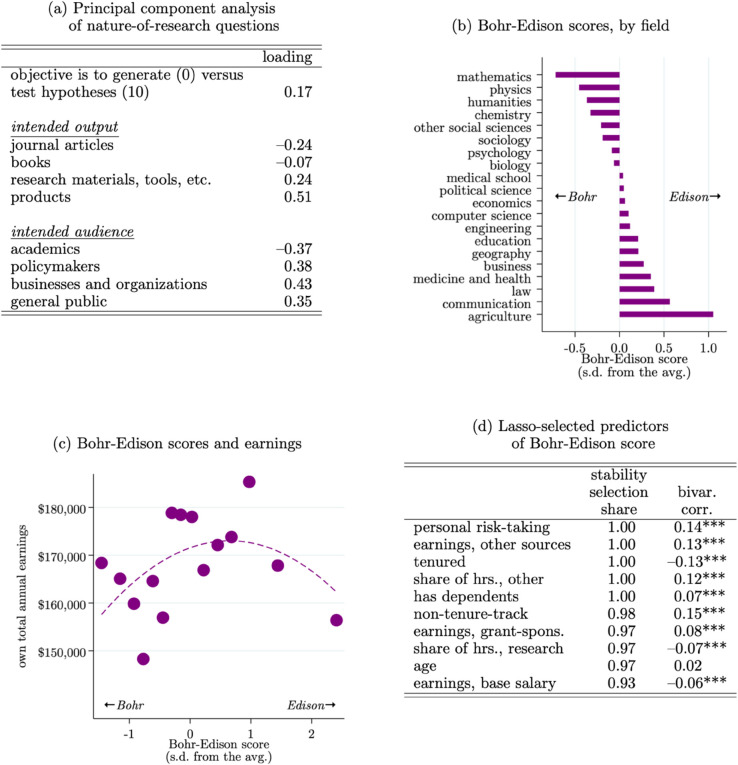
Correlates of the Bohr–Edison (“basic–applied”) spectrum. *Note*: Based on 4,186 observations reporting non-zero research hours. Panel (a) reports results from the Principal Component Analysis (PCA) used to generate the Bohr-Edison score; more positive values indicate more applied, science, and more negative values indicate more basic, science. Panels (b-c) show field-level averages and earnings relationship, respectively. Panel (c) fits a quadratic line; the linear correlation is $0.01^{**}$. Panel (d) reports the results from lasso regressions predicting the Bohr-Edison score with the full set of covariates from the sample; the stability selection share column reports the share of 100 bootstrap sub-samples that the covariate is selected in, and the bivariate correlation column reports that correlation for the full sample; stars indicate the significance:   *p*<0.1, ^**^ *p*<0.05, ^***^ *p*<0.01.

To combine the information contained in all of these nature-of-research questions, we use Principal Components Analysis (PCA) to estimate a single-dimension, standardized index. Fig 7 Panel (a) reports the results from the PCA. On one end of this spectrum are professors focused on generating hypotheses and writing journal articles for academics, and on the other end of this spectrum are professors focused on testing hypotheses and making tools and products for policymakers, businesses, and the general public. Hence, in the spirit of [97]’s [97] quadrants, we term this uni-dimensional index the “Bohr-Edison” score. More negative values indicate more “Bohr”-like basic science and more positive values indicate more “Edison”-like applied science.

Fig 7 Panel (b) reports the field-level average Bohr-Edison scores. The ranking is intuitive, with traditionally “theoretical” fields like mathematics and physics scoring more towards the Bohr end of the spectrum, and more applied and technically-oriented fields like agriculture, law, and medicine scoring more towards the Edison end of the spectrum.

One way to conceptualize the basic-applied spectrum through the lens of economics is the ability of the researcher to appropriate the value of their outputs. The application of more basic research is fundamental in nature and may be harder to appropriate, and vice versa as the research becomes more applied. In this vein, Fig 7 Panel (c) plots professors’ earnings as a function of their Bohr-Edison score. As expected, we find a strong positive correlation. The correlation reverses at the extreme Edison-end of the spectrum, which may be driven by the fact that some of the most applied professors in the sample come from fields such as education, communication, and agriculture, (Fig 7 Panel b) which are fields with some of the lowest average earnings (Fig 1 Panel b).

To understand what variables are most predictive of the Bohr-Edison score, we again use the stability selection approach. Fig 7 (d) reports the top 10 predictors per their stability selection share along with their bivariate correlations with the Bohr-Edison score. A noteworthy finding here is that personal risk-taking is one of the best predictors of doing more applied, Edison-like work. This is an example of a pattern that would be difficult to find using ex-post measures of risk aversion (e.g., based on citations or text of publications and patents) because it is difficult to know whether cross-field differences in such measures are due to underlying professor characteristics or simply reflect field differences (e.g., different citation norms). To the extent the Bohr-Edison score reflects Edison-like entrepreneurship, this finding echoes other work showing that entrepreneurs tend to be more risk tolerant [98–100]. More generally, this approach to transforming researchers’ intentions in terms of the outputs and audience of their science could prove useful in generating observable, ex-ante variation in researchers’ positions along the basic-applied spectrum.

## 4 Discussion

The emergence of large, curated datasets derived from publication and grant records has facilitated a surge of new empirical studies of science. However, there are many important variables that even high-quality administrative datasets do not capture [101]. In this paper, we document our survey efforts to solicit and codify some of these important but hard-to-observe variables for the academic research workforce: professors’ time allocations, their earnings sources, the nature of their research, and their risk aversion. This new survey, combined with existing datasets, yields new insights into variation amidst US academic researchers both *within* and *across* fields at a national scale.

We are certainly not the first to use survey methods to learn about the academic research workforce. But our approach provides one of the first broad views across the full spectrum of science in modern research universities. We do not report any causal effects here, and we are limited in our ability to precisely disentangle sources of heterogeneity across researchers in many dimensions. But we have highlighted a number of novel features of this workforce.

We take a narrow view of the broader academic research workforce here, focusing only on professors. The dramatic rise in contingent and part-time faculty [102] and prevalence of “staff scientists” [103] at US universities suggest that we are clearly missing some important workers. Targeting these researchers would likely require alternative outreach techniques and new survey instruments, but would certainly be worthwhile. Future survey work can build on our efforts more broadly by investing more resources into recruiting respondents, eliciting preferences with more precise methods, or eliciting a wider spectrum of preferences. Such efforts can continue to provide a complementary view of the science of science.

## Supporting information

S1 Appendix Inclusivity in Global Research Questionnaire. (PDF)
