# Supplementary material for: The national survey of academic researchers: New facts and data
Source: PLoS One. 2026 Feb 19;21(2):e0340642. doi: 10.1371/journal.pone.0340642 (PMC12919802; doi:10.1371/journal.pone.0340642)
Supplement: S1 Appendix — (PDF) [file pone.0340642.s001.pdf]

A Additional information on methods

A.1 Population data collection

Our process for the population data collection was as follows. We hired workers to collect publicly available contact information. The workers received detailed instructions on the layout of institutions' websites, the required fields, and data formatting guidelines. After a first round of data collection, the research team manually checked each submitted file by navigating to the relevant institutions' webpage and using random spot-checking to ensure the collected information was complete, accurate, and correctly formatted. If discrepancies were identified, workers were then instructed to add any missing information to ensure a complete record. This process repeated as many times as necessary to complete each file. Finally, the research team cleaned the files to ensure consistent formatting.

A.2 Survey instrument

Below is an abbreviated version of the survey instrument, including the wording of questions and response formats. Some components of the survey not referenced in this paper, as well as other guidance, conditional logic requirements, and notes provided to respondents, are not shown here. Thus, not all respondents were shown all of the following questions.

1. Which of the following best describes your current tenure status or applicability?
    - On tenure track, not tenured
    - Tenured
    - Not on tenure track or tenure does not apply
2. In what year do you expect to be evaluated for tenure at your primary institution?
    - 2022 (this year)
    - 2023 (in about 1 year)
    - 2024 (in about 2 years)
    - ... (continue in this format)
    - 2033 or after
3. In what year did you first receive tenure at any institution?
    - 2022

- 2021
- 2020
- ... (continue in this format)
- 1965 or earlier

4. In what year do you expect your current contract or appointment with your primary institution to be reviewed or evaluated?
   - 2022 (this year)
   - 2023 (in about 1 year)
   - 2024 (in about 2 years)
   - ... (continue in this format)
   - 2033 or after
5. Approximately how often are your contracts or appointments with your primary institution reviewed or evaluated?
   - Every year
   - Every 2 years
   - Every 3 years
   - ... (continue in this format)
   - Every 10 or more years
6. Over the next 5 years, what is your best guess as to how many hours per week you will work on average? (Please try to include any time spent actively engaged in your research, teaching, administration, or effort towards any other positions you hold.)
   - 10 hours or less per week
   - 11-15 hours per week
   - 16-20 hours per week
   - ... (continue in this format)
   - More than 90 hours per week

7. Over the next 5 years, what is your best guess as to how your work time will be spent across the following categories in percentage terms? (Please enter a number between 0 and 100 for each category. Choose the category that best describes the work. Your answers must sum to 100.)

   - Research (including the supervision of others) : _______
   - Fundraising for your research : _______
   - Teaching or advising (not as a part of your own research) : _______
   - Administration or committee service at your institution : _______
   - Clinical and/or medical practice : _______
   - All other (e.g., professional service, consulting, other) : _______
   - Total : _______

8. Approximately, what do you expect your earnings to be from each of the following sources this year? (Note: Do not count earnings in more than one option; choose the option that best describes the source. Please report a pre-tax estimate. If you are in a "soft money" position or you must fund all or a portion of your salary, please report the amount of your salary you must cover in the "Salary covered by research grants or awards" category.)

   - Primary institution: Guaranteed base salary (e.g., "hard money"): _______
   - Primary institution: Salary covered by research grants or awards (e.g., "soft money") : _______
   - Primary institution: Supplemental teaching or other duties: _______
   - Primary institution: Clinical practice and/or medical practice: _______
   - All other wages or salaries from positions outside your primary institution: _______
   - Each category includes a dropdown menu of dollar amounts in $5,000 increments up to $100,000, then $10,000 increments to $500,000, then a single option for $500,000 and above.

9. Over the next 5 years, approximately how much total research funding are you guaranteed to have from any previous or active funding streams? (Note: Please ignore any indirect or overhead costs and report only funding amounts that you can directly

spend. Include funding remaining from any "start-up" packages, internal awards, external grants, or any other funding that you have already obtained and/or are guaranteed to receive. If in doubt, please take "guaranteed" to imply there is more than a 95% chance you will receive the funding.)

- No guaranteed funding
- $5,000 total (avg. $1.0k per year) or less
- $12,500 total (avg. $2.5k per year)
- $25,000 total (avg. $5.0k per year)
- $37,500 total (avg.$7.5k per year)
- $50,000 total (avg. $10k per year)
- ... (continue in this format)
- $250,000,000 total (avg. $50M per year) or more

10. Over the next 5 years, approximately how much funding do you expect to obtain from new research awards given the amount of fundraising you plan to do? (Note: Please ignore any indirect or overhead costs and do not count any guaranteed funding or awards.)

    - No fundraising expected
    - $5,000 total (avg. $1.0k per year) or less
    - $12,500 total (avg. $2.5k per year)
    - $25,000 total (avg. $5.0k per year)
    - $37,500 total (avg. $7.5k per year)
    - $50,000 total (avg. $10k per year)
    - ... (continue in this format)
    - $250,000,000 total (avg. $50M per year) or more

11. Use the scale below to rate whether the overall objective of your current research is more (A) to generate new theories and hypotheses, or is more (B) to test existing theories and hypotheses, or is somewhere in between.

    - Slider scale spanning 0[generate theories] – 10[test theories]

12. In general, how risky do you think your current research projects are? (Note: Use this scale from 0 to 10, where 0 means "very safe" and 10 means "very risky")
    - Slider scale spanning 0[very safe] – 10[very risky]
13. In general, how risky do you think your peers think your current research projects are? (Note: Use this scale from 0 to 10, where 0 means your peers think your projects are "very safe" and 10 means "very risky")
    - Slider scale spanning 0[very safe] – 10[very risky]
14. How often are the following items the intended outputs of your work? Never or barely (0); Sometimes (1); Most or all of the time (2)
    - Academic publications in journals or proceedings
    - Books
    - Data, instruments, materials, methods, software, or tools for other researchers
    - Any kind of consumer-oriented or practical application (e.g., products, patents, policies, etc.)
15. How often are the following groups the intended audience of your work? Never or barely (0); Sometimes (1); Most or all of the time (2)
    - Other academic researchers
    - Policymakers, governments, or other public organizations
    - Businesses or other private organizations
    - General public
16. In general, how willing are you to take risks in your personal life? (Note: Use this scale from 0 to 10, where 0 means "completely unwilling" and 10 means "very willing")
    - Slider scale spanning 0[completely unwilling] – 10[very willing]
17. What is your household's total annual income from all sources including salaries, bonuses, investments, etc.? (Note: Include all income received by any persons, including yourself, residing in your home. Please report a pre-tax estimate)
    - Dropdown menu of dollar amounts in $5,000 increments up to $100,000, then $10,000 increments to $200,000, then $25,000 increments up to $500,000, then

$50,000 increments up to $1,000,000, then $250,000 increments up to $3,000,000, then a single option for $3,000,000 and above.

18. What is your age?
    - 19 or younger
    - 20-24
    - ...
    - 75-79
    - 80 or older
    - Prefer not to say
19. What is your gender identity?
    - Female
    - Male
    - Non-binary, genderqueer, or other not listed
    - Prefer not to say
20. What best describes your race and/or ethnicity? (Note: You may choose more than one. These categories are based on the U.S. Census Bureau's definitions.)
    - American Indian or Alaska Native
    - Asian
    - Black or African American
    - Hispanic, Latino, or Spanish
    - Native Hawaiian or Other Pacific Islander
    - White or Caucasian
    - Other, not listed
21. What best describes your U.S. citizenship status?
    - Domestic-born, U.S. citizen
    - Foreign-born, Naturalized U.S. citizen or Legal Permanent Resident

- Foreign-born, non-citizen and non-permanent resident
- Prefer not to say

22. How many generations have you and your direct ancestors lived in the U.S.?
    - 1
    - 2
    - 3
    - 4 or more
    - Prefer not to say

23. What is your relationship or marital status?
    - Single
    - Married or in a domestic partnership
    - Other
    - Prefer not to say

A.3 Population and sample comparisons

A key challenge for all surveys is ensuring representativeness. Here we report three tests of representativeness using data that is observable for both respondents and non-respondents. First, we test for differences in means of the few observable variables extracted from professors' online information: their aggregate field and their rank. Table A1 reports t tests showing both that our respondents includes a slightly larger share of full professors with a relatively equal smaller share of adjunct, clinical or other professors. We also see under-responding from the medical and health sciences relative to all other fields that is sizable; the recruited share in this field is nearly 45% while the respondent share accounts for just under 30%. Our best hypothesis as to this difference is that it's driven in part by the unique structure of medical schools, which account for the vast number of professors in this field. The title of "professor" can often imply notably different duties and positions at medical schools relative to the rest of academia, with professors at medical schools often carrying a significantly larger load of clinical duties. Thus, to some individuals we recruit from these schools, a survey soliciting responses about one's "research" may seem disproportionately irrelevant. This, combined with the relatively less flexible job arrangements given their clinical

duties, may be the cause for this discrepancy. Altogether, this implies that caution should be had when interpreting results for this particular field.

Table A1: Test of differences between recruited and respondent samples

	mean		
	respondents	non-respondents	diff.
assistant	0.27	0.25	0.01
associate	0.23	0.22	0.01
full	0.40	0.35	0.05***
adjunct, clinical, other	0.11	0.18	–0.07***
engineering, math & related sciences	0.17	0.15	0.02***
humanities & related sciences	0.19	0.15	0.04***
medicine & health sciences	0.29	0.44	–0.15***
social sciences	0.15	0.11	0.04***
natural sciences	0.20	0.15	0.05***
N obs.	4,388	127,284	

Note: Based on 131,672 e-mail-based observations in the 50% of the population sampled for recruitment. t test of differences in means; * $p < 0.1$, ** $p < 0.05$, *** $p < 0.01$.

Figure A1 shows this comparison based on data from the National Science Foundation's HERD survey (National Science Foundation 2023), which reports institutional-level data on the total amount of funding flows into all of the institutions in our population (recall, our population was constructed using the HERD, which is why this data is available for the full population).

Figure A1 reports the distributions and regression tests for mean differences in six metrics of institution-level research funding that compare respondents to the full set of researchers invited to participate ("Sample e-mailed"). The distributions overlap to a large degree (see Panel a). We do estimate statistically significant differences in means (see Panel b), but the magnitudes of these differences are all in the range of approximately 4–6%.

Figure A2 reports the results of a similar exercise, instead using individual-level data on researchers' publication output and grant receipts. This data was obtained by performing a fuzzy match of our population (i.e., using names and institution data) to the Dimensions database (Digital Science 2018), which includes disambiguated researcher-level records. We focus on researchers' publications and grants during the twenty years prior (2003-2022) and see very little differences between our respondents and the full set of individuals invited to the survey. The distributions have strong overlap over the full support (see Panel a), and the mean differences are all insignificant and/or smaller than 7.5%.

Figure A1: Recruitment versus completion sample comparison per HERD metrics

(a) Distributions per HERD R&D measures

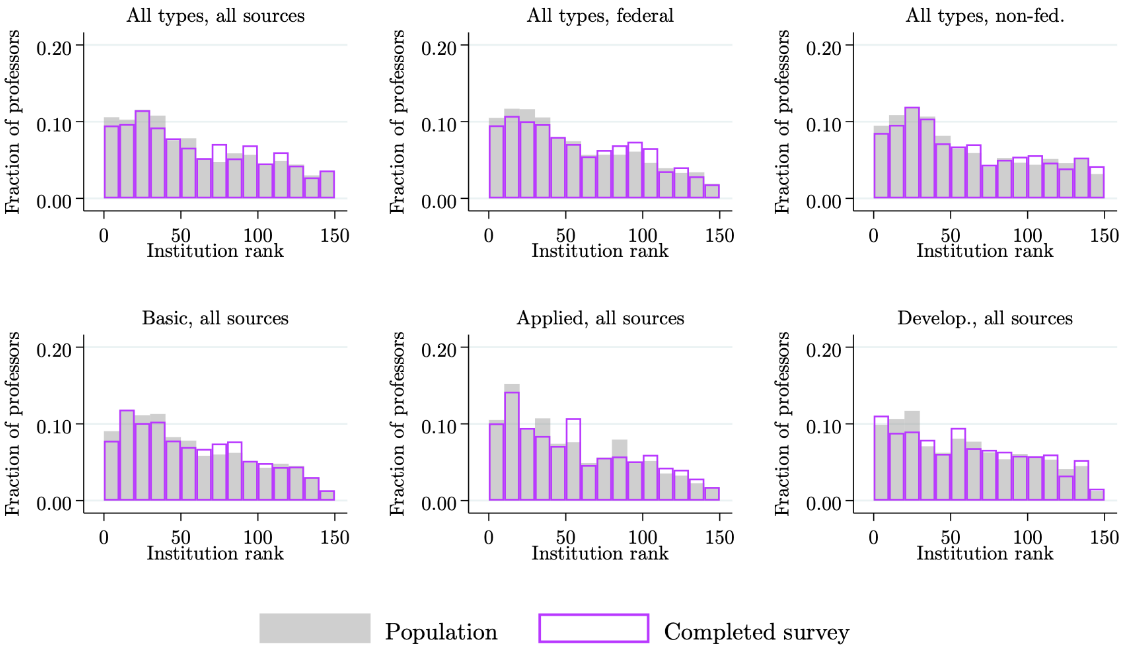

(b) Regression estimates of mean differences

	All sources, by type			All types, by source		
	All	Federal	Non–fed.	Basic	Applied	Develop.
	(1)	(2)	(3)	(4)	(5)	(6)
Completed survey	–35.01***	–21.30***	–13.71***	–24.31***	–7.170***	–3.812***
	(6.953)	(4.511)	(3.062)	(4.867)	(2.492)	(1.328)
Constant	649.1***	351.7***	297.4***	413.2***	176.7***	60.46***
	(1.308)	(0.863)	(0.578)	(0.917)	(0.459)	(0.254)
% diff.	–5.4%	–6.1%	–4.6%	–5.9%	–4.1%	–6.3%
N obs.	130,785	130,785	130,785	130,785	130,735	128,169

Note: Panel (a) compares the distributions of e-mailed professors and respondents per the rank of their institution along each dimension of R&D funding. Panel (b) reports estimates from a regression of each sampled professor's institutional R&D funding (in 2019 $-M) on a dummy for whether the sampled individual completed the survey; the "% diff." row reports the mean difference in the measure as a percentage of the non-respondent average (i.e., it is the ratio of the two coefficients); robust standard errors in parentheses; * $p < 0.1$, ** $p < 0.05$, *** $p < 0.01$. All institutional data is from the 2019 NSF HERD (National Science Foundation 2023).

Figure A2: Recruitment versus completion sample comparison per Dimensions metrics

(a) Distributions per Dimensions publications and grants

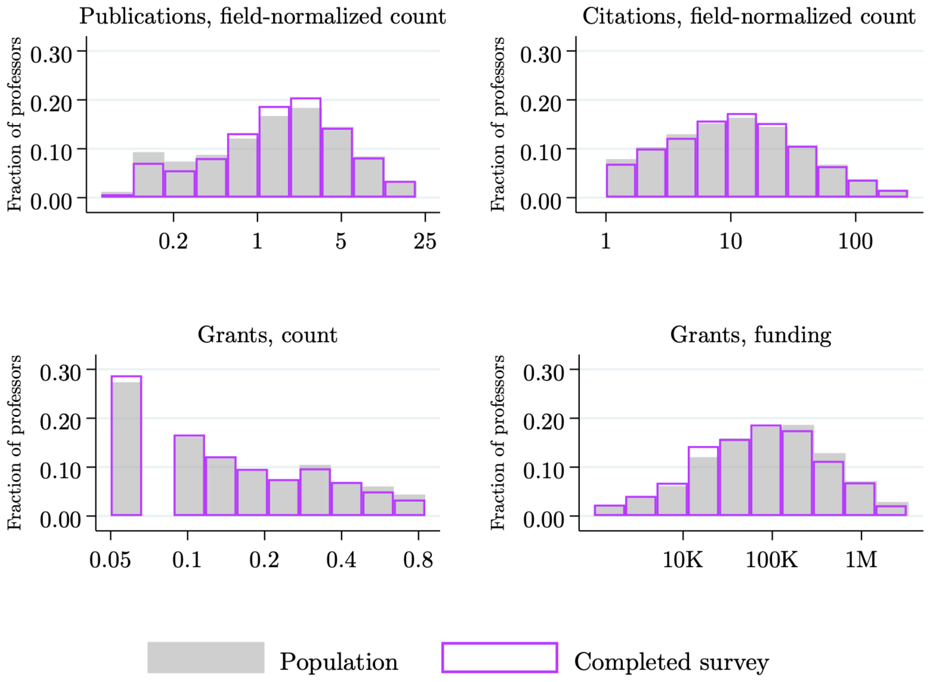

(b) Regression estimates of mean differences

	Pub., count (1)	Pub. cites, normalized (2)	Grant, count (3)	Grant, total $ (4)
Completed survey	–0.111	–0.575	0.00878**	–5568.8
	(0.0688)	(0.807)	(0.00343)	(8519.4)
Constant	2.906***	20.59***	0.117***	121272.4***
	(0.0158)	(0.185)	(0.000715)	(2093.0)
% diff.	–3.8%	–2.8%	7.5%	–4.6%
N obs.	87,000	87,000	87,000	87,000

Note: Panel (a) compares the distributions of e-mailed professors and respondents per each dimension of individual-level publication output and grant funding per year (2003–2022). Panel (b) reports estimates from a regression of each sampled professor's publication or grant metric on a dummy for whether the sampled individual completed the survey; the "% diff." row reports the mean difference in the measure as a percentage of the non-respondent average (i.e., it is the ratio of the two coefficients); robust standard errors in parentheses; * $p < 0.1$, ** $p < 0.05$, *** $p < 0.01$. All publication and grant data is from the Dimensions database (Digital Science 2018).

A.4 Self- and publicly-reported salary comparisons

One limitation of our survey-based methodology is that respondents do not report truthfully due to inattention or some bias such social desirability or an experimenter demand effect. Testing for such issues is notoriously challenging. But one of the survey questions, which solicits respondents' annual salary, provides one test of the degree to which respondents are reporting truthfully since a sub-sample of respondents' salaries are publicly observable.

A number of, primarily publicly owned, institutions in our population publicly post the salaries of their workforce. Using this data we can estimate the extent to which researchers' self- and publicly-reported salaries align.

We used a manual approach to match respondents to their public records (when possible), relying on researchers' names and affiliations to make as high-fidelity of a match as possible. Across and within the 89 institutions that had public salary data, there was a significant amount of heterogeneity in how salaries were reported. In the matching process we made note of any irregularities or confusion for each record matched. Below, we focus only on matches where both we had a high degree of confidence that both (1) the respondent was in fact the individual listed in the public record, and (2) the salary listed represented the full annual salary of that individual.

Figure A3: Self- and publicly-reported salaries

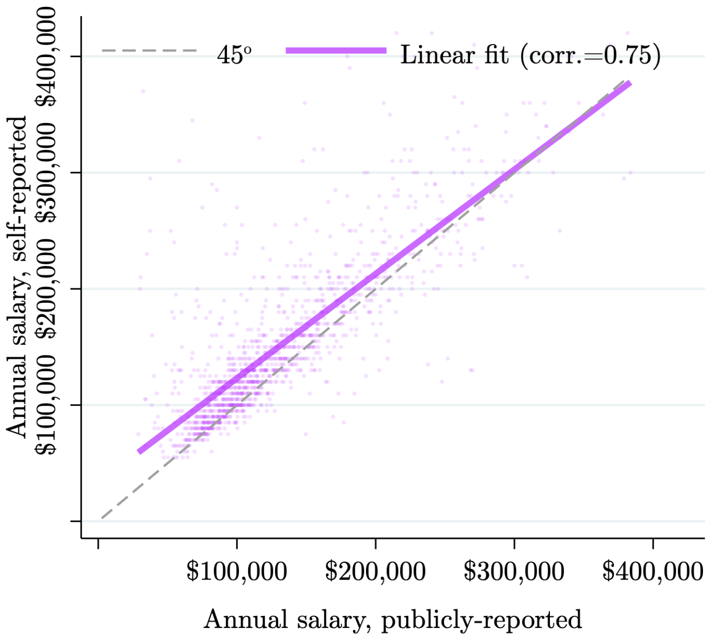

Note: Shows a scatterplot of salaries by source along with a linear fit and 45° line for comparison.

We expect a high degree of alignment between these two measures, but not perfect alignment. Beyond the traditional concern of misreporting by the respondents, misalignment may occur for three other possible reasons: (1) human error in the matching process; (2) the publicly reported salary was for a different fiscal- or calendar-year than what the respondent was reporting or did not in fact reflect individuals' full annual salary; or (3) we solicited salaries in the survey using a pre-populated menu of discrete values to minimize effort, which in turn creates "lumpiness" in the self-reported values.

Figure A3 reports a scatterplot of respondents self- and publicly-reported salaries, which have a correlation of approximately 0.75. Overall, the figure indicates a high degree of alignment between the two measures, suggesting that respondents are answering this question truthfully. Given the traditional sensitivity surrounding salaries amongst this population, especially relative to the questions asked in our thought experiments, this alignment suggests that most respondents were in fact attentive and truthful.

A.5 Narrow and broad fields

Table A2: Field composition of respondents

	type	count	mean	sd
humanities & related				
communication	[0, 1]	4,388	0.02	0.13
education	[0, 1]	4,388	0.02	0.15
humanities	[0, 1]	4,388	0.11	0.31
law	[0, 1]	4,388	0.02	0.14
medicine & health				
medical school	[0, 1]	4,388	0.35	0.48
medicine and health	[0, 1]	4,388	0.08	0.27
natural				
agriculture	[0, 1]	4,388	0.02	0.13
biology	[0, 1]	4,388	0.04	0.20
chemistry	[0, 1]	4,388	0.02	0.13
engineering	[0, 1]	4,388	0.07	0.26
geography	[0, 1]	4,388	0.03	0.17
physics	[0, 1]	4,388	0.02	0.15
social & math				
business	[0, 1]	4,388	0.05	0.22
computer science	[0, 1]	4,388	0.02	0.13
economics	[0, 1]	4,388	0.01	0.12
mathematics	[0, 1]	4,388	0.03	0.17
other social sciences	[0, 1]	4,388	0.02	0.15
political science	[0, 1]	4,388	0.02	0.15
psychology	[0, 1]	4,388	0.02	0.15
sociology	[0, 1]	4,388	0.02	0.14

A.6 Match to grant and publication database

Professors in the full population data were matched to their corresponding records in the Dimensions grant and publication database (Digital Science 2018) using a iterative, fuzzy-merge process based on their name (e.g., transformations and abbreviations thereof) and institution. Within each institution, we limited comparisons to candidates with the same initials (first and last names). For each professor-Dimensions pair, we calculated a fuzzy score (normalized Levenshtein similarity) based on their full names in each dataset. This was implemented with the `fuzzywuzzy` Python library, which computes string similarity scores on a 0–100 scale. The higher of the two scores was used, and matches with a score of 90 or above were retained. The Dimensions disambiguation algorithm errs on the side of representing the same person as multiple records, rather than combining multiple distinct people into one, so when there are multiple matches that meet our criteria we combine them. Overall, roughly 78% of observations are successfully matched.

Table A3 reports t tests using a select set of covariates observable for the full sample to test for differences between the full sample and those professors merged to Dimensions. Clearly, it is not a random sub-sample. Overall, matched professors seem to be more experienced, perform more research, and have higher earnings. This is consistent with the observation (based on our exploration of the Dimensions database) that Dimensions' dismabiguation and record-making processes appear to be positively correlated with research output.

Table A3: Comparison of sub-sample matched to grants and publication data

	mean		
	matched	un-matched	diff.
assistant	0.25	0.24	0.00
associate	0.24	0.30	–0.07***
full	0.43	0.29	0.14***
adjunct, clinical, other	0.08	0.16	–0.08***
engineering, math & related sciences	0.18	0.14	0.03***
humanities & related sciences	0.16	0.28	–0.12***
medicine & health sciences	0.29	0.29	0.00
social sciences	0.17	0.10	0.07***
natural sciences	0.21	0.19	0.02
not on tenure track	0.19	0.32	–0.12***
pre-tenure	0.20	0.23	–0.02*
tenured	0.61	0.46	0.15***
work hours per week	49.11	49.22	–0.11
work-hrs. share, research	0.39	0.31	0.09***
work-hrs. share, fundraising	0.09	0.07	0.02***
work-hrs. share, teaching	0.26	0.34	–0.08***
work-hrs. share, administration	0.15	0.17	–0.01**
work-hrs. share, clinical	0.04	0.05	–0.01**
work-hrs. share, other	0.07	0.07	–0.00
own annual earnings	159,918.75	131,830.99	28,087.76***
earnings share, base salary	0.70	0.71	–0.01
earnings share, grant-sponsored	0.16	0.09	0.07***
earnings share, supplemental	0.03	0.03	–0.01**
earnings share, other	0.04	0.04	–0.00
earnings share, clinical	0.02	0.01	0.00
5-year guaranteed research funding	450,620.67	235,528.17	215,092.51***
5-year fundraising expectations	565,644.75	341,931.92	223,712.82***
N obs.	3,323	1,065	

B Additional summary statistics and results

Table B1: Full-survey one-dimensional PCA, by field

field	aggregate field	count	mean	sd
law	social sciences	71	0.63	2.88
political science	social sciences	165	0.56	1.93
business	social sciences	198	0.29	2.42
economics	social sciences	100	0.27	2.19
psychology	social sciences	177	0.27	1.82
medical school	natural sciences	871	0.21	2.37
chemistry	medicine & health sciences	86	0.20	2.16
physics	natural sciences	159	0.16	2.08
biology	natural sciences	211	0.12	2.36
geography	natural sciences	202	0.07	2.02
sociology	social sciences	155	-0.00	2.03
medicine and health	medicine & health sciences	299	-0.02	2.10
other social sciences	humanities & related sciences	157	-0.11	1.88
engineering	humanities & related sciences	263	-0.31	2.55
agriculture	humanities & related sciences	103	-0.32	1.86
humanities	engineering, math & related sciences	456	-0.33	2.13
communication	engineering, math & related sciences	119	-0.33	2.16
education	engineering, math & related sciences	173	-0.39	2.18
computer science	engineering, math & related sciences	76	-0.54	2.75
mathematics	engineering, math & related sciences	145	-0.69	2.41

Note: Reports one-dimensional PCA score using all relevant survey questions, averaged at the field level, alongside the aggregate field assigned.

Figure B1: Distributions of fields, institutions, and ranks

(a) Field composition of institutions

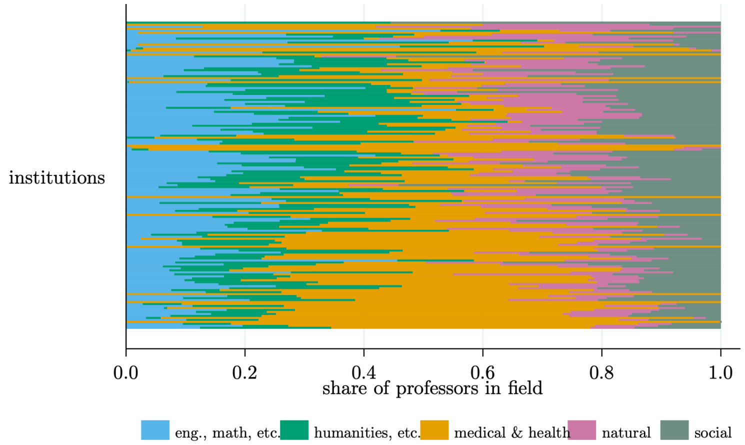

(b) Rank composition of institutions

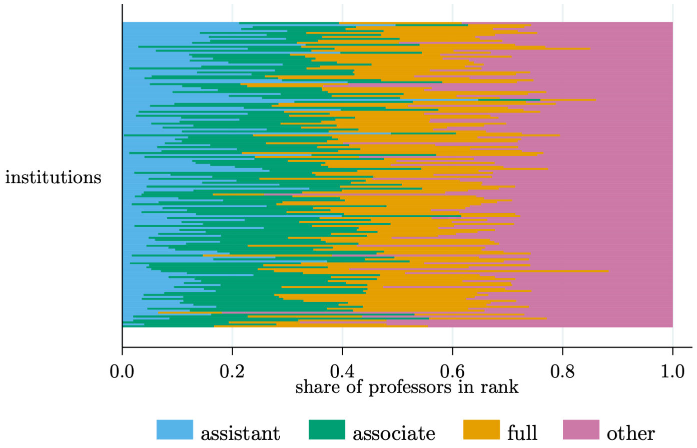

(c) Rank composition of fields

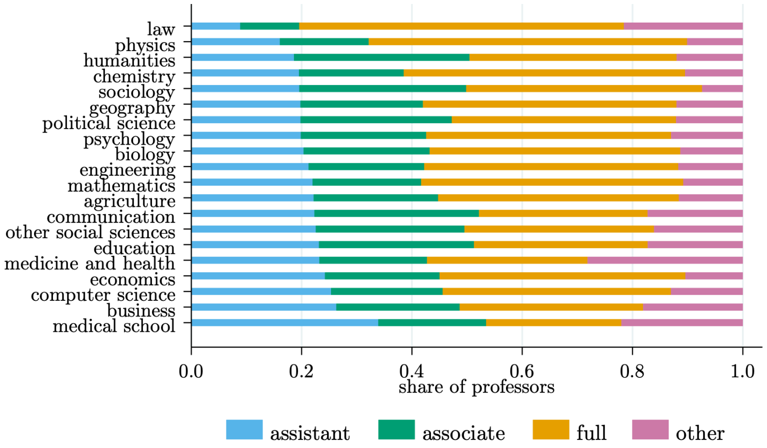

Note: Panels (a–b) plot the share of each institution per field (Panel a) and rank (Panel b); institutions are not labeled, but are sorted by size in terms of number of professors. Panel (c) plots the share of professors in each field by rank.

Figure B2: Time use

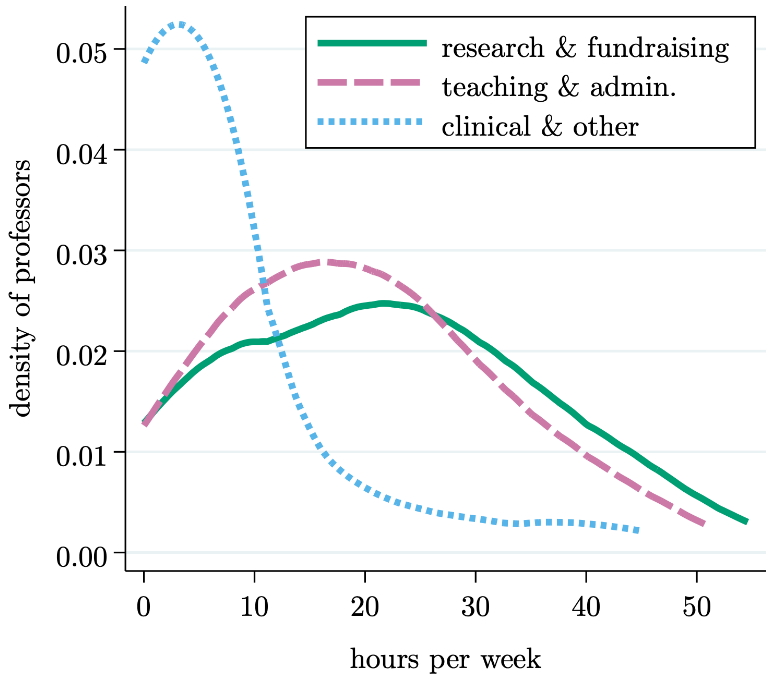

Note: Plots the distribution of professors' expectations of how they will spend their time, on average, each week in the coming five years.

Figure B3: Professors' funding expectations, by aggregate field

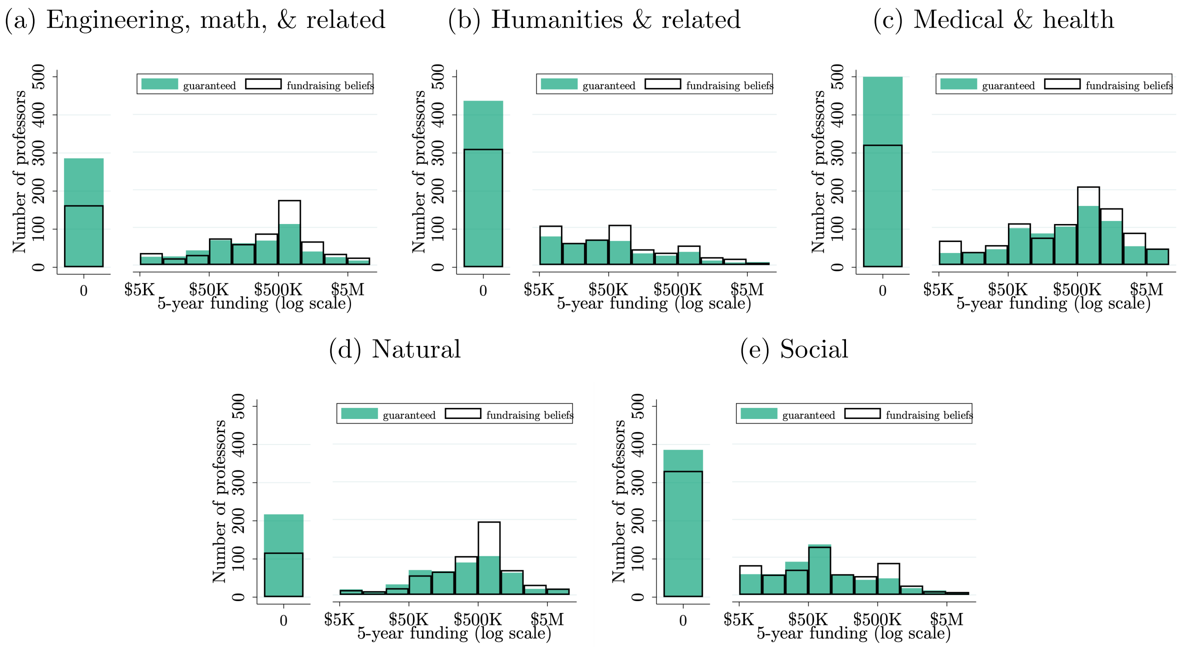

Note: Plots the distribution of professors' research funding in the coming five years based on either funding that is guaranteed (e.g., from prior awards or annual internal funding guarantees) or based on their beliefs about how much funding they will obtain through fundraising (e.g., winning new grants).

Figure B4: Share of earnings sourced via grant-coverage

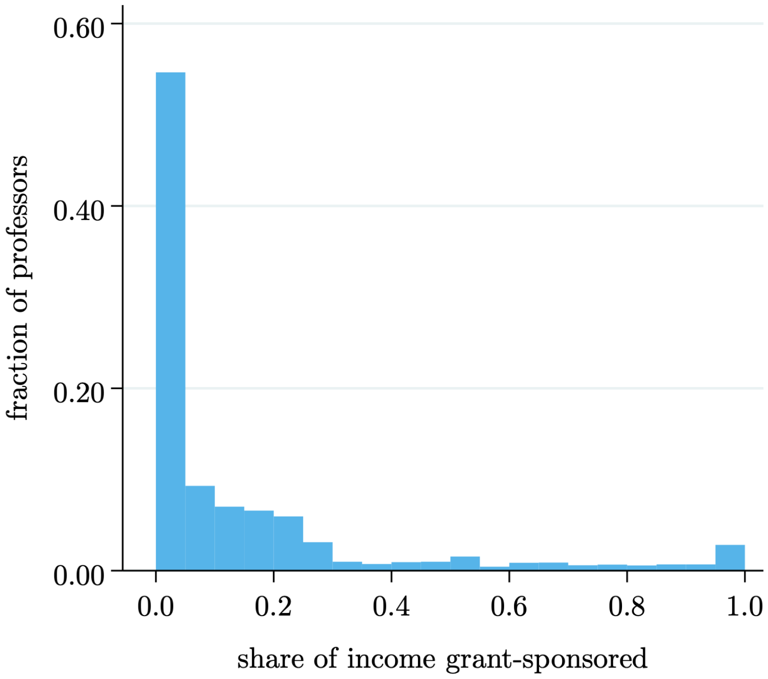

Note: Plots the distribution of the share of professors' earnings that is sourced by grant-sponsorship (e.g., the share of earnings due to "soft-money").

Figure B5: Additional earnings inequality results

(a) Own earnings inequality, by field

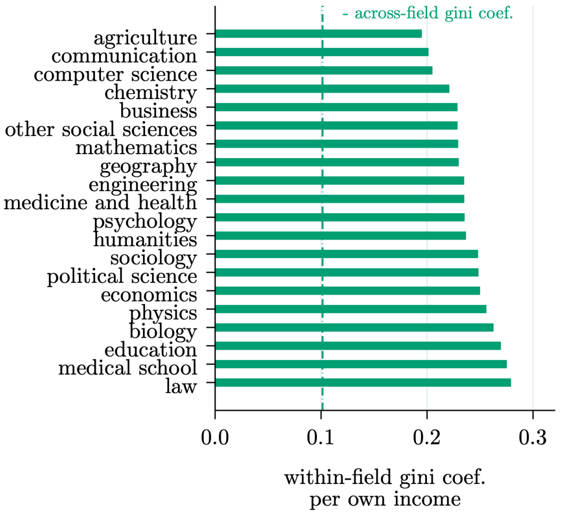

(b) Household earnings inequality, by field

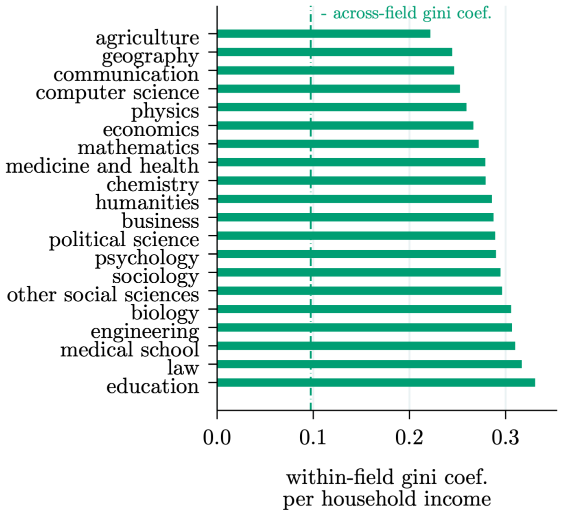

(c) Field level earnings and inequality

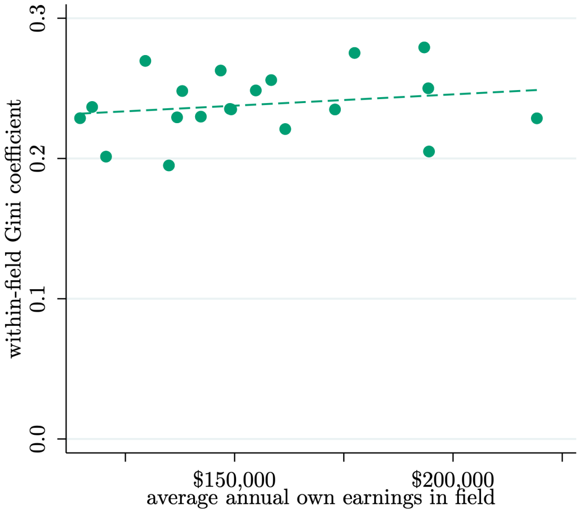

Note: Panels (a–b) plot the within-field Gini coefficients using either own (a) or household (b) earnings. Panel (c) is a scatterplot of within-field Gini coefficients per average own earnings in the same field, showing no significant relationship between the two (i.e., inequality within a field is not correlated with the average earnings in the field); the correlation is $0.20^{(n.s.)}$.

Figure B6: Tasks and earnings sources, by field

(a) Work tasks, share of total

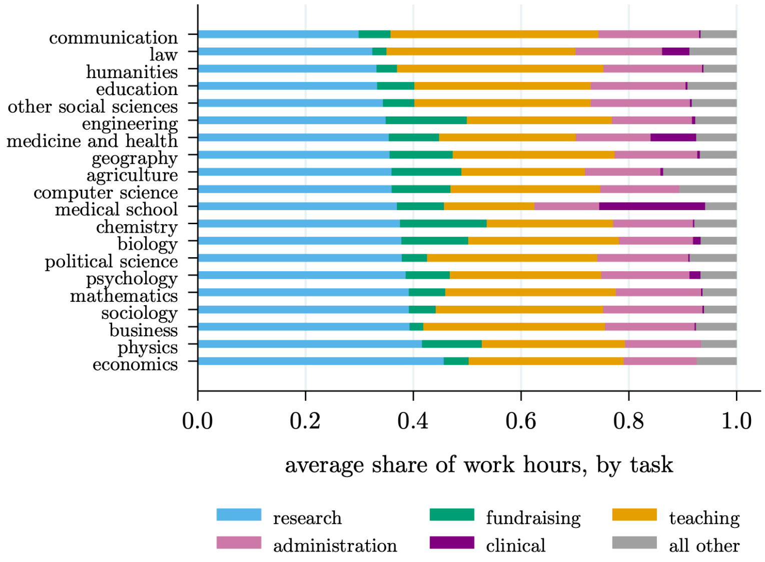

(b) Source of earnings, share of total

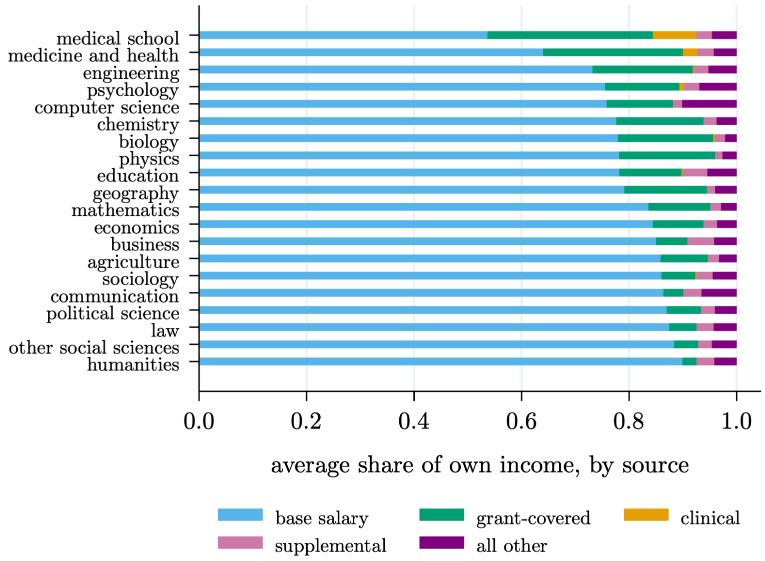

Note: Plots the average share of professors' total work hours spent on each task (Panel a) and the share of their total annual earnings per each source (Panel b) averaged at the field level.

Table B2: Time use correlations

	research	fundraising	teaching	administ.	clinical	other
research	1					
fundraising	0.11***	1				
teaching	–0.24***	–0.12***	1			
administ.	–0.26***	–0.06***	0.06***	1		
clinical	–0.29***	–0.20***	–0.21***	–0.09***	1	
other	–0.12***	0.03*	0.05**	–0.04**	–0.11***	1

Note: Reports the pairwise unconditional correlations in hours worked per week on each task category.

Figure B7: Fundraising productivity and time allocations

(a) Correlation between hourly and annual output

(b) Ratio of hourly and annual output

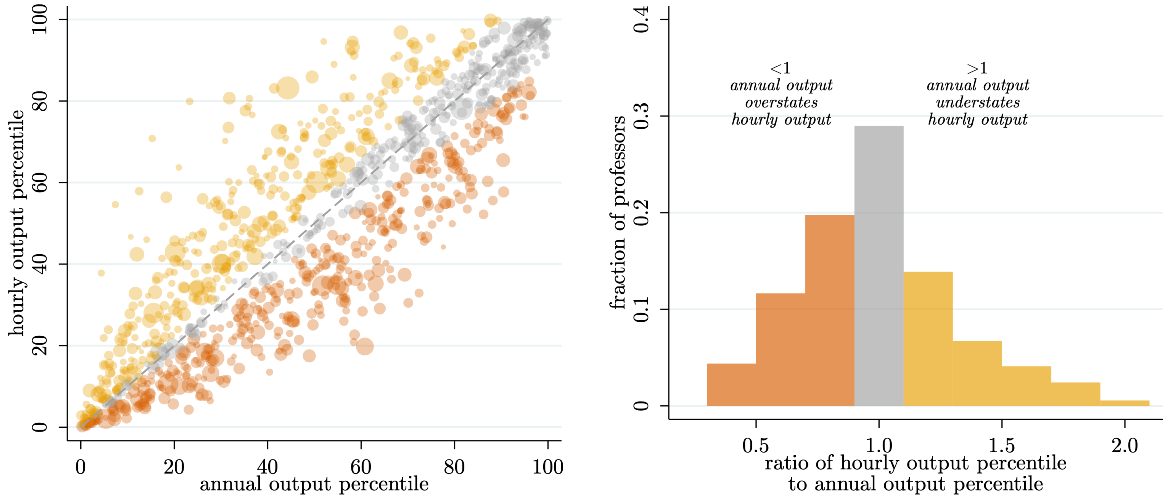

Note: Based on 1,073 observations reporting non-zero fundraising time and matched to the grant database, with the grant database showing non-zero grant dollars. Output is based on field-normalized grant dollars per year or per fundraising-hour.

Table B3: Age and researchers' intended output and audiences

	Outputs				Audience			
	(1)	(2)	(3)	(4)	(5)	(6)	(7)	(8)
Age (decades)	–0.0723***	–0.0568***	0.0503***	0.0920***	–0.00744	–0.0101	0.0318**	–0.00817
	(0.0130)	(0.0126)	(0.0128)	(0.0112)	(0.0127)	(0.0123)	(0.0123)	(0.0124)
Incl. field FE	Y	Y	Y	Y	Y	Y	Y	Y
R^2	0.08	0.11	0.07	0.31	0.11	0.15	0.13	0.12
N obs.	4,083	4,083	4,083	4,083	4,089	4,089	4,089	4,089

Note: Regressions of standardized metrics for researchers' intended output of, and audiences for, their work per their age. All regressions include field fixed effects. See the main text for details on how the intended output and audience scales are constructed.

Table B4: Tenure and researchers' time allocations

	Research, Fundrais.	Teaching	Administration	Clinical, other
	(1)	(2)	(3)	(4)
Post tenure	–4.489***	0.0337	3.748***	0.391
	(0.488)	(0.298)	(0.262)	(0.245)
Incl. field FE	Y	Y	Y	Y
R^2	0.19	0.16	0.10	0.07
N obs.	2,180	2,180	2,180	2,180

Note: Regressions of researchers' time allocations on their tenure status; including tenure-track professors at most 6 year before, or 15 years after, their tenure evaluation. All regressions include field fixed effects.

Figure B8: Distribution of research risk beliefs

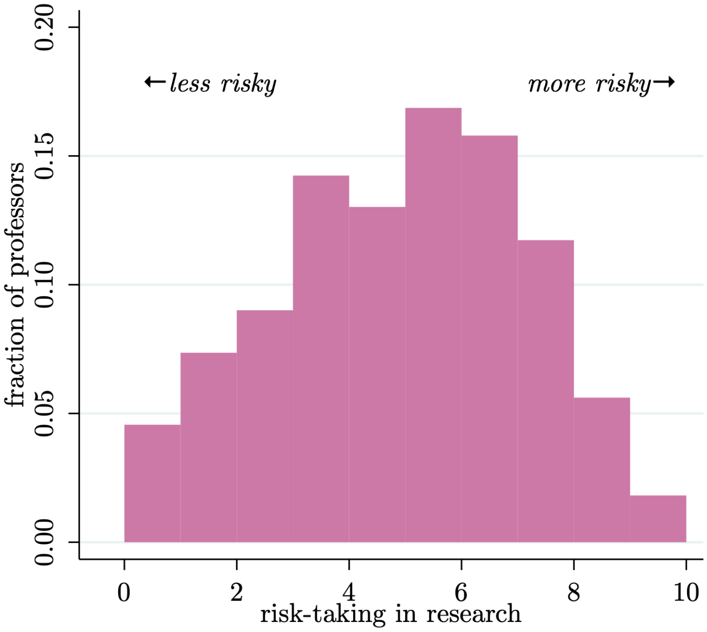

Note: Histogram of the average response to the two questions about researchers' own beliefs and second-order beliefs about peers' beliefs as to the riskiness of their research on a scale from 0 to 10.
